# Introducing “Validated entheses-Based reconstruction of activity 2.0” (VERA 2.0): Semi-automated 3D analysis of bone surface changes

**DOI:** 10.1371/journal.pone.0321479

**Published:** 2025-04-16

**Authors:** Fotios Alexandros Karakostis

**Affiliations:** 1 Palaeoanthropology, Department of Geosciences, University of Tübingen, Tübingen, Germany; 2 Senckenberg Centre for Human Evolution and Palaeoenvironment, Senckenberg Research Institute, Tübingen, Germany; 3 Integrative Prehistory and Archaeological Science, University of Basel, Basel, Switzerland; Tokai University, School of Medicine, JAPAN

## Abstract

In archaeological sciences, the macroscopic morphology of distinct dry bone structures, such as tubercles, ridges, epicondyles, and fossae, is routinely used to infer habitual activity patterns in past human populations, extinct hominins, and other animals. This study introduces “Validated Entheses-based Reconstruction of Activity 2.0” (VERA 2.0), a new method for precisely quantifying 3D surface irregularities on enthesis-bearing bone structures. Building on VERA 1.0, first introduced by the same author in 2016 and later named in a 2021 literature review, VERA 2.0 enhances the previous approach by incorporating a semi-automated image segmentation technique that reduces manual input while maintaining accuracy. The method involves selecting a predefined broad bone surface region, after which an algorithm automatically detects subtle surface irregularities (see example video in the step-by-step protocol at dx.doi.org/10.17504/protocols.io.5jyl82z8dl2w/v3). Validation analyses confirm VERA 2.0’s precision and reliability for activity reconstruction through intra- and inter-observer repeatability tests, experimental research comparing activity and control laboratory specimens, and analyses of historical human skeletons with extensively detailed long-term occupational data. Moreover, while this anthropological 3D measuring protocol paper cannot and does not aim to analyze the anatomical and histological nature of bone surface irregularities, preliminary anatomical dissection and virtual analysis of a cadaveric thumb enthesis suggest a possible association with attaching muscles and ligaments. Future anatomical and histological research aiming to explore soft-hard tissue interactions could clarify how these identified surface changes exactly relate to the attaching tissues. Overall, VERA 2.0 provides a robust, efficient quantitative tool for inferring activity patterns from skeletal remains, with applications across paleontological, paleoanthropological, and bioarchaeological contexts.

## Introduction

Reconstructing habitual physical activity in the past is a fundamental objective of human evolutionary and bioarchaeological sciences. Central to this inquiry is the significance of visually distinct dry bone features – such as tubercles, ridges, epicondyles, fossae, and sulci – which typically serve as attachment sites for muscles and ligaments and are broadly referred to in the anthropological literature as ‘entheses’ [e.g., see extensive reviews in [1–12]. It is important to emphasize that, within the anthropological context, the simplified term “enthesis” generally denotes the broader functional role of these enthesis-bearing bone structures as described in standard anatomical references [e.g., 3,4,13]. This usage reflects an interpretation based on macroscopically visible dry bone morphology rather than the exact muscle or tendon footprint, which cannot be precisely identified in dry skeletal remains [13].

The quantitative analyses of these skeletal features has been shown to yield valuable insights into past animal behaviors [e.g., 2,7,11,14–20], leading a plethora of bioarchaeological studies to routinely rely on size and shape differences in these bone structures for inferring habitual physical activities and behavioral adaptations among ancient human populations and/or extinct hominins [e.g., 2,3,5,6,21–25]. Despite the conceptual potential of these bone structures for reconstructing past behaviors, many traditional measuring techniques have faced significant criticisms regarding their precision and statistical rigor [1,2,26,27]. For instance, many studies have emphasized the subjective nature and lack of repeatability of some approaches, which can result in inconsistent interpretations of entheseal robusticity [10,27–30]. Additionally, most previous methodologies for reconstructing activity have not received any experimental support from laboratory animal models or extensively documented human skeletal samples [e.g., see discussions in 14–16,31,32]. These shortcomings highlight the broader importance of developing more robust and experimentally supported analytical frameworks in skeletal methods aimed at inferring past activity.

In 2016 [12], I introduced a method to address these key challenges, later termed the “Validated Entheses-based Reconstruction of Activity” (VERA) [3]. For the purposes of this study, it will be referred to as “VERA 1.0”. This approach accurately measures the three-dimensional (3D) surface areas of distinct bone structures commonly associated with muscles or ligaments (i.e., “entheses”) and has demonstrated significant intra- and inter-observer precision, as shown in multiple previous studies involving human and other animal skeletal remains [12,17,20–22,27,33,34]. Notably, VERA 1.0 was the first enthesis-based methodology validated through four laboratory animal studies that encompassed diverse species and activity regimes [14–17], alongside human skeletal samples with uniquely detailed long-term occupational documentation [18,19]. Two recent literature reviews [3,26] formally named this approach “VERA,” emphasizing the original measuring protocols developed in 2016, which have been consistently applied in all related studies since. These reviews, along with other studies, provided comprehensive instructions and illustrations for measuring enthesis-related bone structures, thereby facilitating the methodology’s application across various research contexts [7,17,26,27].

While the traditional VERA 1.0 method represents a significant advancement in virtual anthropology, it has certain key practical limitations. One major issue is the considerable training required for new observers, as the process of delineating the exact borders of distinct bone surface structures (e.g., tubercles) is complex and necessitates examining their morphology from multiple angles across their perimeter. For new observers to achieve measurement repeatability, substantial time investment and multiple trials under the direct guidance of the developer (Karakostis) are often necessary, depending also on the observer’s experience with virtual anthropology. This complexity also impacts the speed of delineation, particularly for intricate cases of entheses, thereby prolonging the time required to analyze large skeletal samples. Additionally, VERA 1.0 focuses on delineating the entire supporting bone structure associated with a soft tissue’s enthesis, such as the entire tubercle on top of which a tissue attaches, rather than targeting the direct footprint of the muscle, tendon, or ligament. While this approach is reasonably grounded in the enthesis-organ concept proposed by Benjamin and McGonagle [35] and further discussed in previous entheseal reviews [e.g., 3,16,18,26] – which posit that force and muscle loading during contraction are not confined to the exact footprint but dissipate across surrounding structures – recent findings from a study on lab mice indicated that specific key areas of bone structures presumably associated with the muscle’s footprint (or at least a predominant part of it), such as the most central area of the elevated ridge on top of the deltoid tuberosity, exhibited enhanced signals of physical activity [16]. This suggests that analyzing surface alterations (“entheseal changes”) within such centrally located regions of enthesis-bearing structures may likely yield more valuable insights into reconstructing repetitive and intense muscle loading.

In this context, the present article serves as the validation paper for VERA 2.0, an innovative approach that builds upon the foundational work established by VERA 1.0. This new protocol (S1 File) introduces a streamlined, semi-automated method for the three-dimensional quantification and analysis of distinct dry bone structures (e.g., tubercles and ridges), focusing on surface deformations and irregularities proposed to be more closely associated with the effects of muscle or ligament loading [e.g., see 5,10,16,36,37]. VERA 2.0 simplifies the measuring process by requiring only a single initial manual step to select a broader region of bone surrounding the enthesis-bearing structure in question (e.g., the main body of the tubercle), after which the subsequent delineation and measurement steps can be fully automated. This advancement can significantly reduce measurement times to under two minutes per enthesis following initial training (see the example measurement video provided in the published protocol at protocols.io), making it easier for researchers to analyze large skeletal samples in a shorter timeframe. The comprehensive experimental results presented in this paper suggest that VERA 2.0 effectively identifies activity-related differences, maintaining the robust capabilities of its predecessor while offering a faster and more user-friendly option for researchers. The detailed step-by-step protocol (S1 File) for VERA 2.0 is now available at protocols.io (dx.doi.org/10.17504/protocols.io.5jyl82z8dl2w/v3 [38]), encouraging researchers to adopt this enhanced method in their investigations of habitual physical activity in skeletal samples.

## Materials and methods

The step-by-step protocol outlined in this validation study is published on protocols.io (dx.doi.org/10.17504/protocols.io.5jyl82z8dl2w/v3 [38]) and provides comprehensive information on essential requirements and considerations to keep in mind before implementing the method. Key points are concisely emphasized in the paragraphs below.

When selecting muscle or ligament entheses for analysis using VERA 2.0, it is important to focus on attachment sites previously reported to exhibit projecting surface deformations and irregularities, commonly referred to in the anthropological literature as “entheseal changes”. In cases where there is insufficient literature regarding a specific enthesis-bearing bone structure and its potential surface changes, it is advisable to visually inspect its morphology across the sample under study to identify any consistent irregularities or deformations that cannot be plausibly attributed to taphonomic damage or pathological lesions. If, however, an enthesis-bearing structure consistently displays smooth and unaltered surfaces across all individuals within a sample, the traditional VERA 1.0 method, which relies on overall 3D area measurement rather than targeting specific entheseal changes within it, may be more suitable for its analysis.

In studies focused on reconstructing habitual muscle or ligament use based on the morphology of enthesis-bearing bone structures, the presence of considerably sized pathological lesions or taphonomic alterations can significantly impact measurement reliability. Ideally, bony structures with lesions exceeding 4 mm in diameter should be excluded from the analysis to ensure reliable results (in line with [9,36,39]). However, if it is deemed necessary to retain such an enthesis in a study due to the specimen’s unique importance (e.g., representing a rare fossil hominin specimen) or suspected connections between its enthesopathy and activity-induced biomechanical stress [40], it is crucial to document and discuss these alterations in the study. Furthermore, when dealing with specimens that present such large lesions only within the broader selection region surrounding the enthesis (but clearly outside the main bony structure under analysis), users may proceed with VERA 2.0 while ensuring that affected areas are manually deselected before measurement. For smaller lesions below the 4 mm threshold (either within or outside the main structure), the treatment guidelines outlined in the associated step-by-step protocol (S1 File) [38] should be followed to maintain measurement integrity.

The 3D surface model for analysis can be generated using various technologies, including laser scanning, structured light, computed tomography, or photogrammetry, with the PLY file format recommended (though OBJ and STL formats are also compatible). For this protocol, it is essential to download and use the open-access software Meshlab Version 2023.12 (IST-CNR, Pisa, Italy) or later, as earlier versions lack certain necessary features. Users who choose different 3D image processing software must ensure that all protocol steps and equivalent 3D filtering algorithms are available. It is vital to confirm that the resolution of the surface model is adequate for visualizing surface changes, with a minimum recommended resolution of 300 microns based on trial tests, though a resolution of 200 microns or less is highly recommended. Furthermore, all 3D models in the sample should maintain a consistent (or at least highly comparable) resolution. If significant discrepancies arise (for example, from using different scanning devices or parameters), it is advisable to standardize the resolution using mesh resampling or reconstruction techniques. This can be achieved through standard mesh simplification procedures available in Meshlab, such as “Uniform mesh resampling” (e.g., with a selected absolute precision of 0.05 or 0.10) for all specimens or “Screened Poisson surface reconstruction” (with pre-cleaning). Consistent resolution is crucial to avoid inconsistencies in identifying and measuring entheseal changes. If users encounter any difficulties in homogenizing the resolution of a sample, they are encouraged to reach out to the developer/author directly via e-mail for assistance.

**Fig 1:**
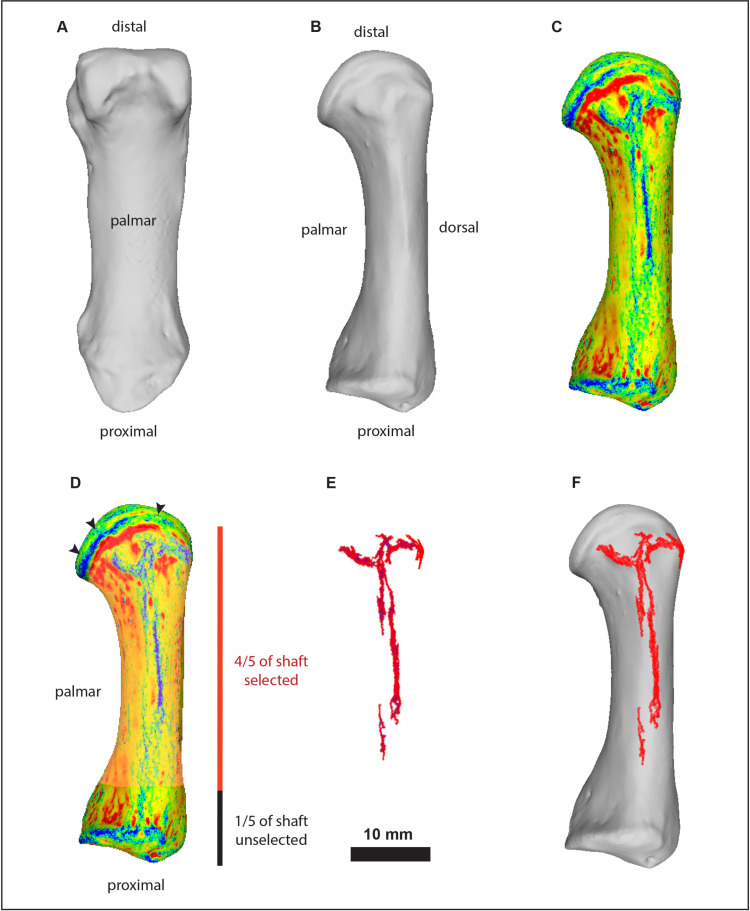
Summary of the steps for delineating surface irregularities within the first metacarpal bone region (right side) associated with muscle *opponens pollicis* (OP), utilizing the semi-automated VERA 2.0 protocol and the stored filter script. (A) Opening the 3D surface model; (B) Rotating the bone to the predefined selection perspective (SP) for this enthesis-bearing structure, which involves positioning the lateral aspect of the bone upright with the distal end facing up; (C) Colorizing the surface using the discrete curvatures algorithm, for observation of potentially pathological or taphonomic lesions. Blue areas indicate high mean curvature (ridges and crests), green areas represent regions of low or near-zero curvature (relatively flat or transitional surfaces), and red areas denote negative Gaussian curvature (saddle-shaped regions) [e.g., 41]); (D) Manually selecting the broader selection region (BSR) with Meshlab’s marker tool, ensuring the selection encompasses 4/5 of the shaft while excluding the articular surfaces (indicated by the three upper black arrows); (E) Applying the stored filter script (*MLX* file in Meshlab) to segment the projecting surface changes; (F) Optionally overlaying the resulting areas onto the original 3D model of the bone for observation and potential corrections.

**Fig 2:**
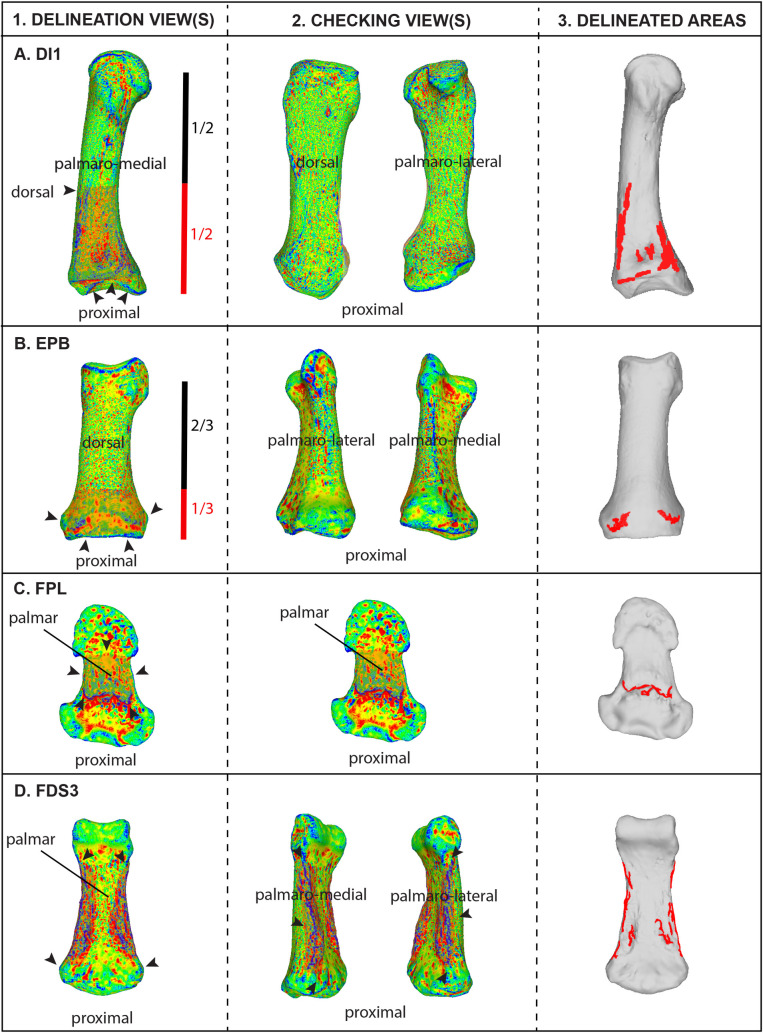
Summary of the VERA 2.0 delineation process for four other enthesis-bearing bone regions in the human hand, commonly associated with muscles of major evolutionary and functional importance. (A) dorsal *interosseus* 1 (DI1) on the first metacarpal; (B) *extensor pollicis brevis* (EPB) on the first proximal phalanx; (C) *flexor pollicis longus* (FPL) on the first distal phalanx; (D) *flexor digitorum superficialis* (FDS) on the third intermediate phalanx. For each enthesis, the figure illustrates: (1) the delineation view associated with its selection perspective; (2) the additional “checking views” used to ensure that no areas were accidentally selected away from the intended broader selection region (BSR); and (3) the resulting delineated surface changes, overlaying the original 3D models, following the application of the filter script. Exact recommendations for defining the selection perspective (SP) and BSR for these entheses are provided in protocols.io (dx.doi.org/10.17504/protocols.io.5jyl82z8dl2w/v3). These examples serve as a foundation for future analyses of the same bone regions and for studying other enthesis-bearing structures with comparable morphology and location. They also offer a basis for researchers to develop their own SP and BSR for other entheses not covered in the protocol and validation paper. The depicted 3D models have been colorized using the discrete curvatures algorithm: Blue areas indicate high mean curvature (ridges and crests), green areas represent regions of low or near-zero curvature (relatively flat or transitional surfaces), and red areas denote negative Gaussian curvature (saddle-shaped regions) [e.g., 41].

**Fig 3:**
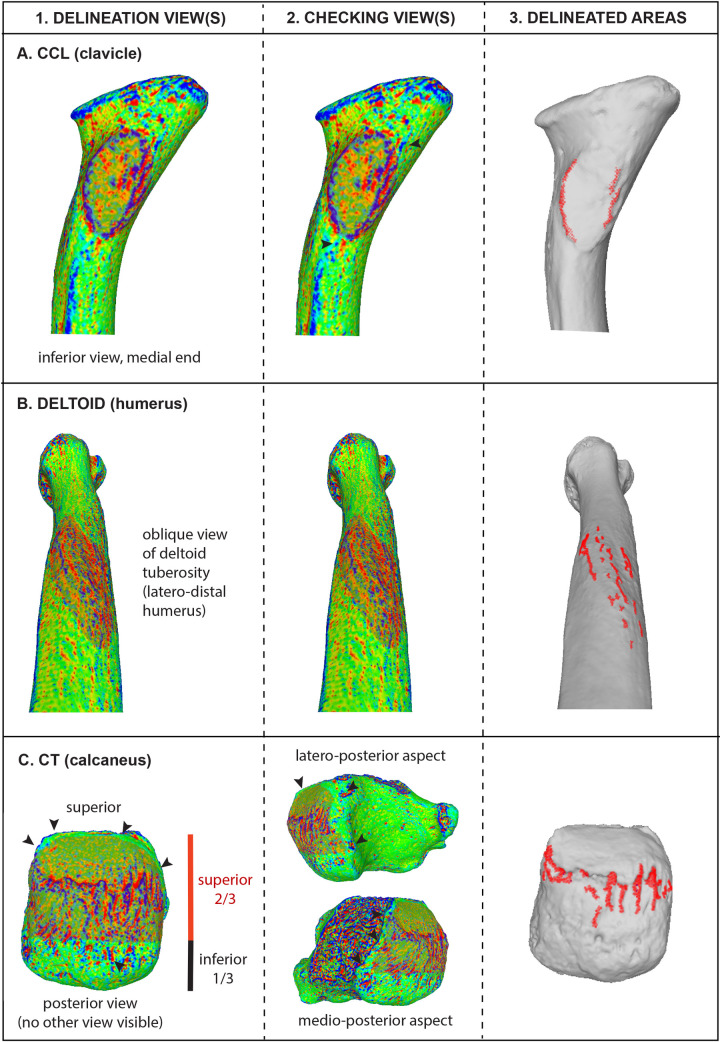
Summary of the VERA 2.0 delineation process for three other enthesis-bearing bone structures of the human skeleton, corresponding to three widely studied and functionally important soft tissues. (A) the costoclavicular ligament (CCL) of the clavicle; (B) the deltoid muscle on the humerus (specifically, the deltoid tuberosity); and (C) the Achilles tendon on the calcaneus (specifically, the calcaneal tuberosity, here defined as “CT”). For each bone region of focus, the figure illustrates: (1) the delineation view associated with its selection perspective; (2) the additional “checking views” used to ensure that no areas were accidentally selected away from the intended broader selection region (BSR); and (3) the resulting delineated deformed areas overlaying the original 3D models following the application of the filter script. Exact recommendations for defining the selection perspective (SP) and BSR for these entheses are provided in protocols.io (dx.doi.org/10.17504/protocols.io.5jyl82z8dl2w/v3). These examples serve as a foundation for future analyses of these structures and for studying other enthesis-bearing regions in the skeleton with comparable morphology and location. They also provide a basis for researchers to develop their own SP and BSR for other entheses not covered in the protocol and validation paper.

As described in the Introduction, the only essential manual task in VERA 2.0 is defining a broader selection region (BSR) that encompasses a significant portion of the bone surface surrounding the bone structure under analysis (see examples for different cases of entheses in Figs 1–3). This selected region must be consistent across the sample for the same enthesis, based on anatomical orientation and traits. To ensure this consistency, a specific selection perspective (SP) of the bone is defined for each enthesis across the entire sample, mainly guided by anatomical orientation. The process of defining SP and BSR is visually enhanced through the use of Meshlab’s discrete curvatures algorithm, which colorizes the surface (following [41]). This algorithm helps distinguish between relative projections (in blue shades) and depressions (in red shades), making it easier to visually discern outlier “island” projections around the main attachment structure (e.g., the tubercle), but still within the defined BSR. In cases where such isolated blue-colored structures appear to represent pathological or taphonomic lesions (based on standard anthropological criteria), they should be treated as explained in the paragraph above (as well as in the protocol itself, in more detail). Additionally, the discrete curvatures filter helps avoid the accidental selection of articular surface rims (typically represented as distinctive blue lines on the model) and any written labeling on the bone surface, both of which should be excluded from the BSR. Detailed instructions for this process are available in the provided steps and delineation example video on protocols.io. The steps of the protocol include specific additional recommendations for nine example entheses of the human skeleton, such as rotating the bone again to confirm that no excessive areas were accidentally selected away from the BSR (refer to “checking views” in Figs 2 and 3). These examples serve as a foundation for defining the BSR for these and other entheses with similar morphology and bone locations, while also assisting researchers in developing their own selection perspectives (SP) and broader selection regions (BSR) for other entheses of interest. In this paper, Figs 1–2 provide examples of the SP and BSR for eight of these examples, while the protocol also includes detailed information and illustrations regarding the broad metacarpal bone region commonly associated with the insertion of the muscle *opponens digiti minimi*, which requires a few additional considerations.

After defining the BSR, the remaining measurement process can be fully automated using a stored filter algorithm script (in Meshlab, this is an *MLX* format file). An example script is available for download, editing (if necessary), and reuse on protocols.io, with an example video demonstrating its application. This fast process, driven by the filter script, is also summarized in Fig 1 below (with more examples provided in Figs 2 and 3). In brief, as detailed in the step-by-step protocol, the filters will slightly smooth the bone surface (reducing small, sharp artifacts; this step should be skipped if the model is already smooth), select the vertices associated with the manually defined BSR, invert the selection to isolate the rest of the bone, and delete the surrounding bone. Then, the script colorizes the remaining (BSR) surface based on computed principal directions of curvature, using the quadric fitting method and the “minimum curvature” criterion, to highlight less pronounced surface features (e.g., see [42]). Subsequently, the color histogram is equalized to homogenize the shades of blue, representing surface projecting irregularities. All projecting areas are then automatically selected and isolated by removing the surrounding bone areas. Small outlier surfaces around the main body of identified surface irregularities are also automatically eliminated (but this step is editable, as explained in the protocol). Finally, the 3D surface area of the irregularities (in blue color shades) is measured in square millimeters.

### Ethics declarations

No experiments or new skeletal materials were generated for this validation study, which exclusively relied on existing and published 3D data (surface models). All the materials utilized in the validation analyses of this study have been previously published and reused in multiple prior studies (as referenced in the text), with the consent of the relevant institutions and in compliance with all international legal and ethical standards. This includes the 3D data from the bioarchaeological skeletal remains (curated in the Natural History Museum of Basel, Switzerland, which provided the scans) as well as the already published and available open-access lab animal 3D data (https://doi.org/10.5061/dryad.ksn02v70p). The latter scans derive from older experiments that had received ethical approval from the Institutional Animal Care and Use Committee of Brown University (see https://doi.org/10.1016/j.jhevol.2017.02.001 and https://doi.org/10.1242/jeb.213058).

### Validation studies

This section concisely presents the thorough analyses performed to validate this new approach in terms of: (a) intra- and inter-observer measuring repeatability; (b) experimental support using a laboratory animal sample (comparing between activity and control specimens); and (c) validation based on human skeletons with a uniquely detailed level of long-term occupational documentation. Additionally, subsection (d) describes a pilot anatomical dissection and virtual analysis of an enthesis using a fresh cadaveric human hand, suggesting that VERA 2.0’s measurements may potentially align with entheseal changes within the precise footprint of attaching soft tissue structures. However, the aim of this anthropological 3D measuring protocol paper was not to explore the exact anatomical and histological nature of these soft-hard tissue interactions. These interesting preliminary findings are only meant to encourage future anatomical and histological research, to better understand the exact etiology of the surface irregularities identified by VERA 2.0.

#### (a) Intra- and inter-observer measuring repeatability.

To confirm that the semi-automated measuring protocol of VERA 2.0 is repeatable within and among different observers (following a thorough and meticulous self-study of all details included in the step-by-step protocol), repeatability tests were conducted on three enthesis-bearing bone structures of major functional importance that correspond to three of the most frequently studied anatomical regions of the postcranial skeleton: the arm (humerus), the hand (thumb metacarpal), and the foot (calcaneus). These consisted of the insertion site of *opponens pollicis* (OP), the deltoid tuberosity, and the calcaneal tuberosity (i.e., the attachment structure of the Achilles tendon, which is often considered as the archetypical fibrocartilaginous enthesis). The intra-observer repetitions were performed by the author, while inter-observer repetitions involved a different observer for each of the three enthesis-bearing bone structures (for more information on the three additional observers, see Acknowledgements). A total of 10 bones were measured in each case, including specimens from both anatomical sides (Table 1). All repetitions were done independently and on different days, while training only included a single demonstration of the protocol, including a clear description of the SP and BSR for these enthesis-bearing structures (Figs 2 and 3; also see detailed information on these entheses in the protocol). Following statistical recommendations for testing precision in entheseal 3D areas [26], repeatability was evaluated using the Lin’s concordance correlation coefficient (Lin's CCC), which showed values of 0.95 or above, reflecting a strong level of repeatability and reproducibility [43,44]. Additionally, a series of paired t-tests found no significant differences (P >  0.05) between repetitions within the same observer (the author) and across observers. Despite the high repeatability observed in this study, as noted in previous methodological work on VERA 1.0 [17,27], morphological complexity varies significantly across different enthesis-bearing bone structures of the body, inevitably leading to different levels of difficulty in selecting an identical BSR across individuals. Therefore, I strongly recommend that future users of VERA 2.0 always statistically confirm their measuring repeatability before conducting analyses, as was also suggested for VERA 1.0 [3,26,27]. Users should follow the step-by-step protocol carefully, ensuring that both SP and BSR are as clearly defined and consistent across their sample as possible. If any practical difficulties arise, users are always encouraged to contact the developer for additional guidance or support.

**Table 1:** Results of the repeatability tests.

Enthesis-bearing bone structure	Bone	Intra-observer (Lin’s CCC)	Inter-observer (Lin’s CCC)
*Opponens pollicis* (OP) ridge	Thumb metacarpal	0.99	0.97
Deltoid tuberosity (DT)	Humerus	0.97	0.95
Calcaneal tuberosity (CT)	Calcaneus	0.96	0.95

#### (b) Experimental validation using laboratory animals.

An original analysis was conducted to evaluate the reliability of the VERA 2.0 method for reconstructing habitual activity based on the morphological comparison of enthesis-bearing bone structures. The sample consisted of 15 laboratory-raised Eastern wild turkeys, all female and one-year-old, previously analyzed in a study employing the traditional VERA 1.0 approach [14]. During the experiment, turkeys were divided into three groups: uphill runners, downhill runners, and controls, with treadmill running over 10 weeks (at a speed of 2.5 m s^ − 1^, for 30 min a day, 4 days a week). The original VERA 1.0 analysis had identified clear patterns in three distinctive enthesis-bearing structures of the femur (associated with muscles *gluteus primus*, medial *gastrocnemius*, *vastus medialis*, and *adductor magnus*), clearly distinguishing runners from controls. In this study, I applied VERA 2.0’s semi-automated protocols to the same samples and entheses. Nevertheless, because the birds’ 3D surface models were relatively small and already quite smooth, applying the smoothing step and using a 2 mm threshold for artifact removal consistently resulted in “0” areas being identified across most of the sample’s specimens. Therefore, it was necessary to skip the initial smoothing step and reduce the artifact removal threshold to 1 mm, following the protocol’s recommendations (see S1 File). It is important to note that this issue did not arise in the human skeletal analyses discussed in the next subsection (and shown in Figs 1–3), which also included relatively small hand bones.

**Fig 4:**
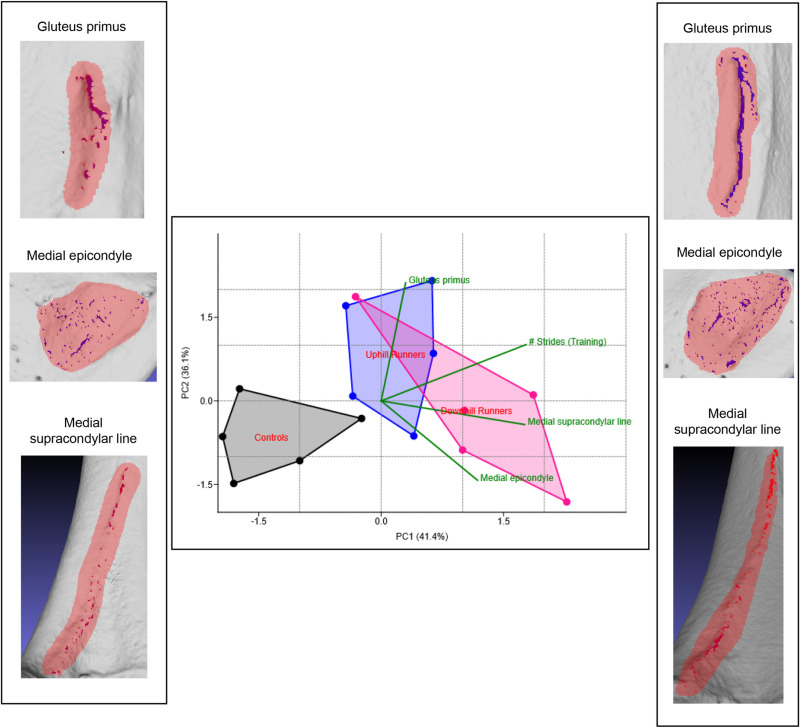
Principal component analysis (PCA) results comparing control turkeys with uphill and downhill runners, based on 3D surface changes on enthesis-bearing structures, measured using VERA 2.0. In the side example images (representing the entheseal patterns of negative and positive PC1 scores, respectively), BSR regions are highlighted in red, with blue areas showing the automatically defined surface irregularities.

Subsequently, a principal component analysis (PCA) and other multivariate models were used to compare the groups (following [26,45]). The results (Fig 4) show clear differences between controls and both groups of runners, along with a trend distinguishing uphill from downhill runners. In the figure’s side images (left and right), examples of delineated irregularities are provided for each enthesis-bearing structure: the BSR is highlighted in red, while the blue areas indicate the entheseal changes identified. These examples illustrate the patterns of controls (negative PC1 scores) versus runners (positive PC1 scores). Importantly, including “strides” (the exact number of steps each bird took) in the PCA revealed a strong covariance between the identified 3D surface changes (in mm^2^) and the exact number of strides performed (Fig 4). Three additional analyses were conducted in R, to explore this pattern [45] (Table 2). A multivariate analysis of variance (MANOVA) comparing groups (without the strides variable) showed significant and very strong differences (Pillai’s Trace =  0.88). A multiple regression model showed that the identified surface changes significantly predicted the number of strides, explaining 52% of their total variance (based on the R^2^ value). Finally, an analysis of covariance (ANCOVA) adjusting for body mass confirmed that the observed entheseal changes were not due to body size differences among birds. For all three analyses, the necessary statistical assumptions were met, including linearity (but no multicollinearity), absence of outliers (in the variables and the residuals), approximate normality of residuals, homogeneity of slopes, homoscedasticity per group, and homogeneity of variance-covariance matrices, in line with standard statistical recommendations [26,43,45,46]. Overall, these results confirm VERA 2.0’s reliability in reconstructing activity in experimental settings, without necessarily needing size adjustment. In total, measuring and analyzing the surfaces of all 45 enthesis-bearing bone structures required one working day.

**Table 2:** Summary of statistical results from three analyses comparing control, uphill, and downhill runner turkeys based on 3D surface changes on enthesis-bearing bone structures, measured using VERA 2.0.

Analysis	Independent Variable	Dependent Variable	F-value	P-value	Effect Size Measures (Pillai’s Trace, R^2^, Partial η^2^)
MANOVA	Activity groups (3)	3D surface irregularities on all three enthesis-bearing structures (3)	2.89	**0.031**	0.88
Multiple Regression	3D surface irregularities on all three enthesis-bearing structures (3)	Number of strides performed	3.95	**0.039**	0.52
ANCOVA (**covariate** = bird mass in grams)	Activity groups (3)	PC1 scores	8.50	** < 0.01**	0.61

This includes results from the multivariate analysis of variance (MANOVA), multiple regression predicting number of strides from entheseal changes, and analysis of covariance (ANCOVA) adjusting for body mass.

#### (c) Validation using documented human skeletons.

To further validate the reliability of the VERA 2.0 protocols for reconstructing physical activity in humans, I conducted a study using anthropological remains from the 19th-century Basel-Spitalfriedhof collection, curated by the Natural History Museum of Basel, Switzerland [18,47,48]. This collection is uniquely valuable for biological anthropology due to its rare, detailed documentation on each individual’s occupation, duration of employment, occupational changes during life, and exact work hierarchy [18,47]. Previous studies using VERA 1.0 demonstrated clear distinctions between occupational activity levels in biological males, separating heavy manual laborers (mainly construction workers, including stonemasons, carpenters, and bricklayers) from precision workers engaged in low-intensity tasks (e.g., tailors, shoemakers, and painters) [18]. Similarly, research on biological females from the same collection identified distinct patterns in females with specific occupations (“FSO”), such as trained tailors and seamstresses, compared to those with more generalized tasks (e.g., housemaids and factory workers without specifically archived job descriptions) [19].

In this validation study, I re-measured selective key enthesis-bearing bone regions from these individuals using VERA 2.0’s semi-automated process. The resulting measurements and exact specimen labels are available open-access in the Zenodo repository (https://doi.org/10.5281/zenodo.15000406). All necessary permits were obtained for the described study from the responsible institution (Natural History Museum of Basel, Switzerland), which complied with all relevant regulations.

**Fig 5:**
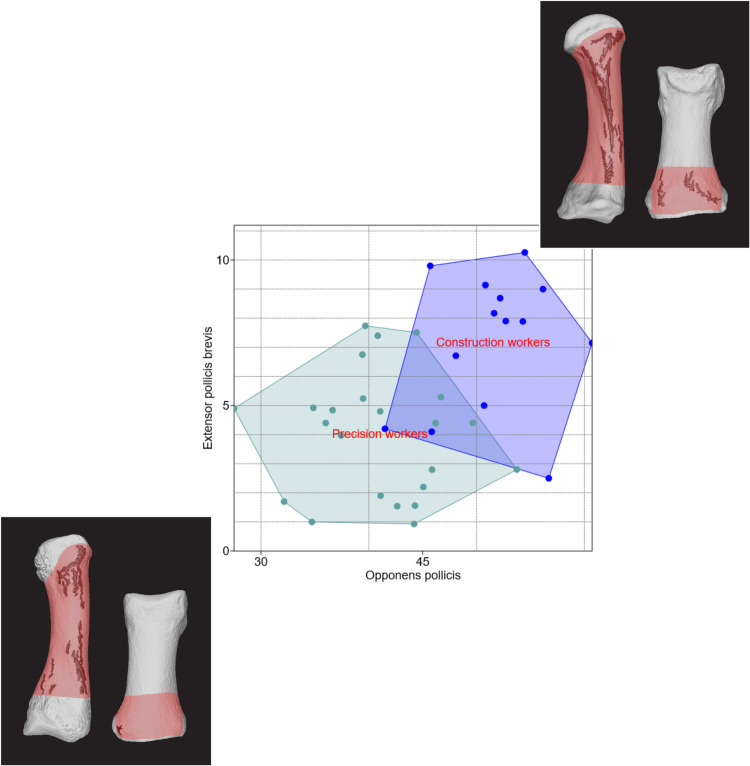
Bivariate plot showing variation between biological male construction workers (heavy manual laborers) and precision workers, based on 3D surface regularities within bone regions associated with muscles *opponens pollicis* (OP) and *extensor pollicis brevis* (EPB). Larger surface irregularities were observed in construction workers, highlighting their increased biomechanical stress due to manual labor. Side images show examples of the BSR (highlighted in red) and surface irregularities (in blue) for each occupational group.

**Fig 6:**
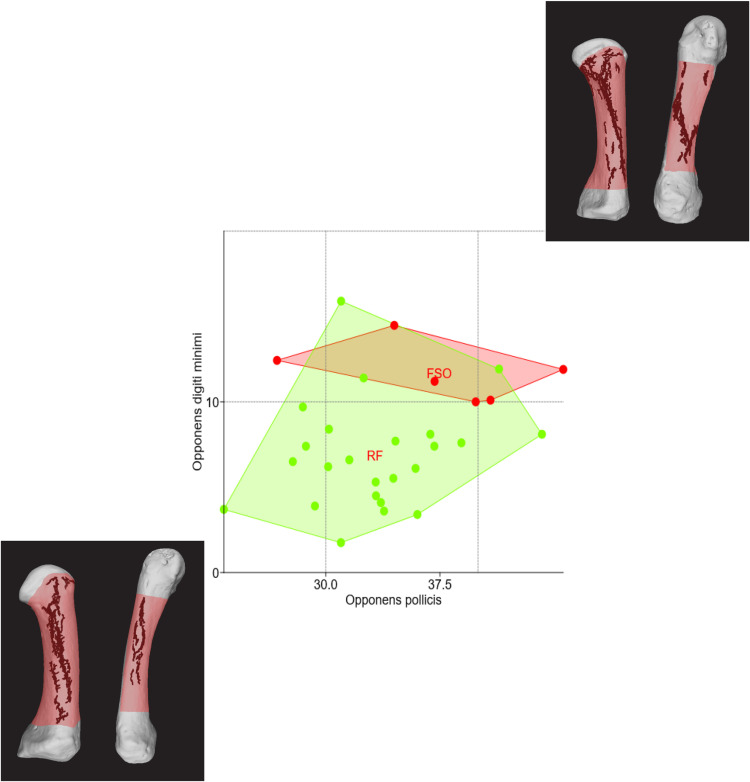
Bivariate plot showing the distinction between female workers with specifically defined occupations (FSOs) and generalized workers (mainly maids and factory workers), based on 3D surface regularities within bone regions associated with muscles *opponens pollicis* (OP) and *opponens digiti minimi* (ODM). FSOs exhibited overall larger surface irregularities, likely reflecting more biomechanical stress in occupational tasks requiring coordination of the thumb and the little finger via opposition (in line with previous results and activity interpretations regarding these individuals [19]). Side images show examples of the BSR (red) and surface irregularities (blue) for each group.

Without applying any size adjustments, Fig 5 shows that simply plotting the 3D surface size of irregularities within bone regions associated with two muscles – OP and *extensor pollicis brevis* – on a bivariate XY plot visually separates long-term construction workers from precision workers. This separation represents entheses of muscles associated with thumb extension and opposition. The figure includes side images of each occupational group’s enthesis-bearing bone regions, with SP and BSR highlighted in red and surface irregularities in blue. Similarly, the female plot (Fig 6) of two enthesis-bearing bone regions (related with muscles OP and *opponens digiti minimi*) demonstrated a clear separation between FSOs and those with generalized tasks. FSOs showed significantly larger 3D changes in both regions, which represent muscles used for thumb and little finger opposition. To statistically support these findings, I additionally performed a MANOVA to compare between occupational trends in each case (males and females). Significant differences between occupational groups (P <  0.05) were found in both sexes (Table 3), while all assumptions for MANOVA were met [26,43,45,46]. Spearman’s correlation tests were also conducted to evaluate the relationship between the observed surface irregularities and factors such as biological age and body size (using femoral head diameters [49]), finding no significant correlations (P > 0.05) in this well-documented sample [26].

**Table 3:** Multivariate analysis of variance (MANOVA) results comparing the two occupational groups within each biological sex.

Biological Sex	Analysis	Independent Variable	Dependent Variable	F-value	P-value	Effect Size (Pillai’s Trace)
**Males**	MANOVA	Activity groups (2)	3D surface irregularities on both enthesis-bearing structures (OP, EPB)	22.81	** < 0.01**	0.57
**Females**	MANOVA	Activity groups (2)	3D surface irregularities on both enthesis-bearing structures (OP, ODM)	7.28	** < 0.01**	0.35

Males: construction workers vs. precision workers; females: individuals with specifically defined occupations vs. more generalized workers.

Interestingly, both male heavy manual laborers and FSOs tended to display significantly larger surface irregularities in both attachment sites analyzed, a result different from VERA 1.0, which found distinct occupational differences in the proportions among different enthesis-bearing structures (rather than larger raw values in one of the groups compared). This is probably because VERA 1.0 quantifies the entire supporting bone structures associated with an enthesis (e.g., the 3D surface of the entire tubercle within which the tendon footprint is located), whereas VERA 2.0 aims to filter out discrete surface irregularities (“changes”) within that area (see discussion in [16]). Based on the literature, the extent of these localized bone alterations is presumably more dependent on the amount of biomechanical stress directly applied on the surface by the attaching soft tissue [1,4,5,10,13,14,16,50] compared to the bone remodeling responses that affect the gradual uniform change of the entire supporting bone structure (e.g., the tubercle) [9]. This scenario would explain why the more strenuous occupational trends within males or females presented overall greater changes in both enthesis-bearing structures analyzed (for the strenuous manual activities of FSO individuals, see discussion in [19]), which was also the case for the habitual runners of the experimental animal dataset (Fig 4). It would also explain why, in contrast to VERA 1.0’s raw measurements (prior to adjustment), VERA 2.0’s raw measurements do not significantly correlate with body size or age and can directly distinguish occupational trends without requiring size adjustment (at least in the datasets of the present study). This is because, in case these entheseal surface irregularities are primarily stress-inflicted, they would not be expected to be as correlated with body size as larger bone structures that first form during development [3,26,12]).

However, this does not mean that analyzing multivariate proportions among enthesis-bearing structures is irrelevant for VERA 2.0 data. Different activity patterns engage various muscles, and those used more frequently are expected to exert greater loading onto their associated bone regions (e.g., as seen in the varying clarity of occupational differences between OP and the more distinct *opponens digiti minimi* in Fig 6). This proportional approach may better distinguish occupational trends than analyzing individual enthesis-bearing bone structures in isolation [3,15,12]. Thus, the integrative univariate and multivariate statistical analyses outlined in VERA 1.0’s formal protocol [26] remain highly recommended for VERA 2.0 measurements.

#### (d) A pilot anatomical examination and future research possibilities.

The presence of various surface irregularities on enthesis-bearing bone structures, often simplified in the anthropological literature as “entheseal changes”, has been traditionally associated with cumulative biomechanical loading inflicted by the attaching muscles, tendons, or ligaments (e.g., [1,2,5,8,10]). Multiple anatomical and clinical studies have studied these traits from a histological perspective, explicitly addressing the factors driving the appearance of bony projections within entheseal footprints (i.e., bony spurs, lipping, and exostoses) [35–37,13,51–53]. However, no research has utilized fresh cadaveric dissections to verify the exact correspondence between soft tissue and the specific types of scored ‘entheseal changes’ commonly analyzed in anthropological research to infer physical activity in bioarchaeological contexts (i.e., ranked stages of relative robusticity in entheseal bony projections; see [10,39,50,54]). This question is also relevant to VERA 2.0, which automatically identifies and quantifies subtle projections within enthesis-bearing bone structures, even though it uses an initial BSR that includes a much larger portion of the bone (e.g., Fig 1 and example delineation video at protocols.io). According to all analyses and trials conducted in this validation study (see Figs 1–6 and their example illustrations), which involved numerous individuals and diverse species, VERA 2.0’s automatically identified surface irregularities were always predominantly occurring within the enthesis-bearing bone structure under analysis (e.g., within the body of the targeted tubercles or ridges).

Although the analyses of subsections b and c support VERA 2.0’s ability to distinguish among different activity groups, similar to previous experimental work with VERA 1.0 (see animal laboratory studies by [14–17]), the precise anatomical basis for its calculated surface changes remains an open question. Future anatomical and histological research is essential to confirm whether (or not) these automatically identified surface irregularities on bone structures correspond to the exact footprint of the soft tissues responsible for these deformations. While this issue falls outside the scope of the present 3D morphometric study, which explicitly focuses on introducing an efficient new tool for quantifying bone surface changes that are already routinely used in bioarchaeological contexts, I have included here a pilot anatomical dissection only as a preliminary step toward addressing this question in the future. This preliminary case-study only aims to lay a first foundation for future investigations into the relationship between 3D surface irregularities and soft tissue attachment, which could potentially enhance the multidisciplinary value of VERA 2.0 beyond bioarchaeological inferences of activity.

**Fig 7:**
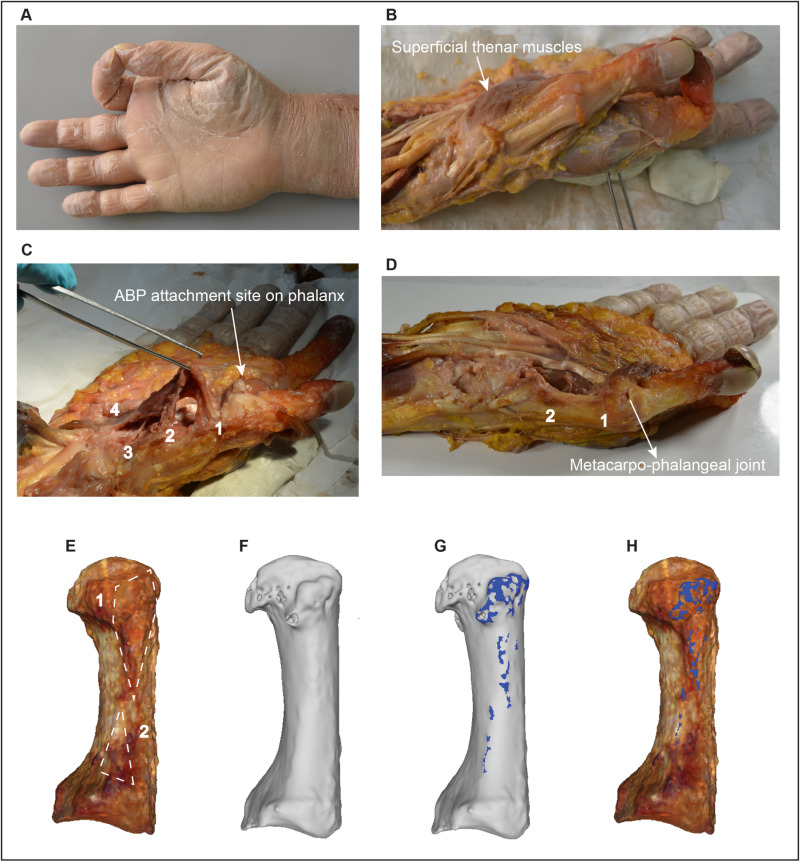
Dissection and virtual analysis of the *opponens pollicis* (OP) muscle’s attachment on the thumb metacarpal (all photographs and pictures of this figure are unpublished and were produced and edited by the author; see Acknowledgements). **(A)** Cadaver hand placed in thumb opposition, **(B)** Exposure of superficial thenar muscles, **(C)** OP musculotendinous unit with attachment origin and insertion points labeled (1-4), **(D)** Thumb metacarpal bone and overlaying fibrocartilaginous layers exposed, **(E)** 3D surface model of the metacarpal’s lateropalmar aspect (distal is up), showing the brown-looking thick fibrocartilage layers within OP’s insertion regions (note: the two approximate main insertion areas are outlined), **(F)** Micro-computed tomography 3D model of the bone after virtually removing the soft tissue layers, **(G)** VERA 2.0 detection of entheseal changes (in blue), **(H)** Superimposed VERA 2.0 changes on the bone prior to virtual segmentation (i.e., soft tissue still present). More details are provided in the text. Scale is not respected across panels, but the metacarpal’s maximum length is 45 mm.

This case-study involved the dissection and virtual examination of the right OP’s musculotendinous unit and its insertion on the thumb metacarpal. It was performed using a fresh cadaveric hand from an adult male body donor under 60 years of age, with no manual pathological conditions. The dissection, carried out at the Medical School of Athens, Greece (see Acknowledgements), is illustrated in Fig 7. The hand was first positioned with the thumb and index fingertips brought in contact, to highlight the position of the thumb metacarpal and the thenar musculature involved in thumb opposition (Fig 7A). Once the skin and fat layers were removed, the superficial thenar muscles were exposed (Fig 7B). Further dissection (Fig 7C) revealed OP’s musculotendinous unit, which was manually pulled upwards, identifying four attachment points on the bone (labeled in the panel). These were: (1) a broad insertion site spanning from the metacarpal head to the bone’s midshaft (lateropalmar aspect), (2) a second and smaller insertion site in the proximal metacarpal (lateropalmar aspect), (3) an origin site on the trapezium’s tubercle, and (4) an additional large origin area on the *flexor retinaculum*. After the removal of the OP musculotendinous unit, the underlying bone and fibrocartilaginous layers were exposed (Fig 7D). The metacarpal was then surgically extracted, and a high-resolution 3D surface model was created, including surface texture and color (Fig 7E). This model highlighted the presence of a thick fibrocartilaginous layer (shown in brown color) within insertion areas 1 and 2 (outlined in the panel), which tended to be thicker in insertion area 1 (where both the OP muscle and joint capsular ligament used to attach). Before removal, the attachment of OP covered the predominant part of this area, while the capsular ligament only attached along the distal margins of the fibrocartilaginous layer, near the metacarpal head’s articular surface (as it can be partly observed in Fig 7D). Unfortunately, after the removal of these two soft tissues (see Fig 7E), visually distinguishing between their two exact attachment sites on the continuous and uniform surface of the fibrocartilaginous layer was impossible. Subsequently, micro-computed tomography was used to scan the metacarpal at a resolution of 40 microns. Through volume rendering and segmentation processes (using the software Avizo; Thermo Fisher Scientific, Massachusetts, United States), the fibrocartilaginous layers were virtually removed to reveal the underlying dry metacarpal bone (Fig 7F). Interestingly, before using VERA’s advanced 3D imaging filters, the resulting bone model only exhibited a small bone tubercle beneath the soft tissue layer, and only within the distal half of insertion area 1 (see Fig 7F), revealing that the actual soft tissue attachment region of OP and its supporting fibrocartilaginous layer were substantially larger than the visible underlying bone projection. VERA 2.0 was then applied to the OP’s enthesis, automatically detecting the 3D entheseal changes marked in blue (Fig 7G). These changes largely corresponded to both OP insertion regions (1 and 2) and their associated fibrocartilaginous layers, demonstrating VERA 2.0’s ability to detect subtle surface irregularities not clearly visible to the naked eye (even in high-resolution 3D models). To further highlight this observation, the 3D entheseal changes were virtually superimposed on the original 3D surface model (using Avizo tools), which still included the fibrocartilaginous layer (Fig 7H). This confirmed that the entheseal irregularities detected by VERA 2.0 (within a broadly defined BSR) largely corresponded to irregularities within the actual footprint of the attaching soft tissues. Overall, this analysis demonstrates that VERA 2.0 has the capacity to accurately identify subtle entheseal changes beneath the soft tissue attachment footprints, even when the defined BSR includes a much larger area and regardless of the bone’s visible gross morphology.

As highlighted above, despite these impressive preliminary observations, I consider this subsection's anatomical case-study (dissection) only as the first step in this new line of anatomical and histological research, and I encourage future interdisciplinary studies to study additional entheses of the human body, after seeking and confirming access to larger fresh cadaveric samples of body donors with documented activities and non-pathological musculotendinous units.

Additionally, it is crucial to highlight again that VERA 2.0 could not distinguish between different soft tissue attachments within the same BSR, as exemplified by the OP muscle and joint capsular ligament in this dissection. This limitation was not surprising, given that there was no clear visible distinction between the borders of these two structures on the thick fibrocartilaginous layer (Fig 7E) or the underlying bone surface (Fig 7F), even in this fresh and healthy cadaveric hand with perfectly preserved soft tissue. Despite this limitation (which is likely inherent to all osteological analyses), VERA 2.0’s potential ability to identify bone surface changes related to soft tissue attachments remains highly relevant for inferring lifetime physical activity. In cases where different tissues attach in the same BSR region, observers should recognize and discuss that the detected entheseal changes may reflect the combined biomechanical influences (stress) of all structures involved. This does not necessarily impede the nature or value of functional interpretations (as previously discussed [19,12,21,23]). For example, humanlike thumb opposition routinely involves both OP contraction and strong flexion of the first metacarpophalangeal joint, to which the capsular ligament directly contributes [55–58], suggesting that the stress experienced by these two neighboring structures is hardly uncorrelated (see relevant discussion in [21]). Therefore, while VERA 2.0 (or any other anthropological approach to date) may not be able to automatically isolate individual attachments within a given bone BSR, its results will usually still be functionally meaningful (e.g., Figs 4–6), as neighboring or overlapping soft tissues often tend to closely interact during movement. This principle was consistently demonstrated in previous studies using VERA 1.0, which frequently analyzed enthesis-bearing structures corresponding to more than one muscle, such as for example the lateral basal tubercle of the human thumb’s proximal phalanx, which accommodates the insertion sites of the two synergist thenar muscles *abductor pollicis brevis* and *flexor pollicis brevis* [3,12,13,18,19,21,23–26].

## Expected results

The expected results of VERA 2.0 underscore its versatility in archaeological sciences, offering a semi-automated, powerful, and fast 3D measuring tool for investigating physical activity patterns across various geochronological contexts. In more recent contexts (bioarchaeology), it offers a robust protocol for identifying activity-related stress levels, while its 3D measurements can also contribute to the analysis of nonhuman faunal remains, as demonstrated in previous VERA 1.0 work [14–17,34], providing researchers with tools to identify potential patterns of domestication and animal use [34]. Additionally, in extinct fossil species where the exact locations of muscle or ligaments are entirely unknown, VERA 2.0’s segmentations could theoretically assist in identifying broader enthesis-bearing structures, thus suggesting an approximate position of muscles or ligaments when direct soft tissue evidence is unavailable. Compared to its predecessor (VERA 1.0), VERA 2.0 is easier to learn, faster to apply, and does not seem to require size adjustments when inferring activity patterns, although future research is needed to explore the potential multidimensional effects of body size in other samples and enthesis-bearing bone structures, particularly within high weight-bearing anatomical regions. Moreover, while VERA 2.0 is primarily designed for bone surface analysis, its ability to semi-automatically distinguish and quantify subtle deformations or irregularities on surfaces offers promising potential for other research areas, such as studying external characteristics of pathological bone lesions (e.g., arthritis or trauma) or stone tool modifications.

## Supporting information

S1 File Step-by-step protocol, also available on protocols.io: dx.doi.org/10.17504/protocols.io.5jyl82z8dl2w/v3. (PDF)
