# Supplementary material for: Introducing “Validated entheses-Based reconstruction of activity 2.0” (VERA 2.0): Semi-automated 3D analysis of bone surface changes
Source: PLoS One. 2025 Apr 16;20(4):e0321479. doi: 10.1371/journal.pone.0321479 (PMC12002543; doi:10.1371/journal.pone.0321479)
Supplement: S1 File — (PDF) [file pone.0321479.s001.pdf]

protocols.io

Mar 07, 2025 Version 3

Protocol for the ‘Validated Entheses-based Reconstruction of Activity 2.0’ (VERA 2.0) Method: Semi-Automated Measurement of 3D Entheseal Changes V.3

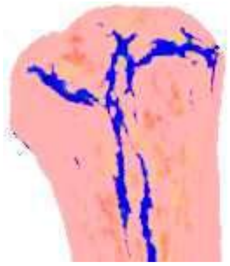

DOI
dx.doi.org/10.17504/protocols.io.5jyl82z8dl2w/v3

Fotios Alexandros Karakostis[1]

[1]University of Tuebingen, Senckenberg Research Institute, University of Basel

VERA 2.0

Fotios Alexandros Karakostis
University of Tuebingen & Senckenberg Research Institute &, ...

OPEN ACCESS

DOI: dx.doi.org/10.17504/protocols.io.5jyl82z8dl2w/v3

Protocol Citation: Fotios Alexandros Karakostis 2025. Protocol for the ‘Validated Entheses-based Reconstruction of Activity 2.0’ (VERA 2.0) Method: Semi-Automated Measurement of 3D Entheseal Changes. **protocols.io** **https://dx.doi.org/10.17504/protocols.io.5jyl82z8dl2w/v3**Version created by **Fotios Alexandros Karakostis**

License: This is an open access protocol distributed under the terms of the **Creative Commons Attribution License**, which permits unrestricted use, distribution, and reproduction in any medium, provided the original author and source are credited

Protocol status: Working
We use this protocol and it's working

Created: September 20, 2024

Last Modified: March 07, 2025

Protocol Integer ID: 123987

Keywords: Entheseal changes, phenotypic variation, biocultural evolution, Validated Entheses-based Reconstruction of Activity (V.E.R.A.), virtual anthropology

Abstract

This protocol introduces the 'Validated Entheses-based Reconstruction of Activity 2.0' (VERA 2.0), an advanced iteration of the original VERA 1.0, which was developed by the same author of this protocol in 2016 (Karakostis & Lorenzo, 2016) and later named in two subsequent literature reviews (Karakostis, 2022; Karakostis & Harvati, 2021). VERA 1.0 has become a valuable tool in anthropological studies for repeatably delineating, measuring, and analyzing 3D muscle attachment sites (surface areas), demonstrating that entheseal 3D surface variation can reflect habitual muscle coordination and physical activity. VERA 2.0 builds on this foundation with significant enhancements, offering a semi-automated protocol that greatly simplifies the 3D area quantification process. It requires only one initial set of manual steps – selecting a pre-specified broader region of the bone around the enthesis – after which the remaining procedure can be fully automated using a stored filter script (e.g., *mlx* file in the software Meshlab). After some practice, this semi-automation can potentially reduce the entire 3D area measurement time to less than 2 minutes per enthesis (see example video below), enabling the analysis of very large skeletal samples within a few working days. Unlike VERA 1.0, which measures the entire 3D surface area of bony structures associated with a muscle and/or ligament (e.g., the entire tubercle), VERA 2.0 identifies 3D surface deformations and irregularities within the footprint of the attaching soft tissue (i.e., "entheseal changes"), which can arguably be more clearly associated with direct muscle or ligament loading. Extensive experimental analyses, which are presented in the validation paper associated with this protocol, indicate that VERA 2.0 can effectively and repeatably identify activity-related differences, similar to VERA 1.0.

This protocol provides a comprehensive, step-by-step guide for executing the VERA 2.0 workflow, covering both manual and automated components. It is designed to enable users to achieve measurement repeatability without necessarily requiring training from the developer, though direct contact with the author is encouraged for questions or explicit recommendations regarding particular entheses.

Guidelines

1. Some sections ("Parts") start with an introductory “**Note**” inserted within the first step right before the description of the step itself.

2. Occasionally, important “**Notes**” are also included within steps, where necessary or useful.

3. Software options or commands are usually indicated within parenthesis (e.g., XYZ → XYZ → XYZ).

4. **IMPORTANT:** The entire analytical process of this protocol (Parts 1 to 4) is described in detail only for the insertion site of muscle *opponens pollicis* (OP) on the right first metacarpal bone. For Part 2, which focuses on the manual component of this protocol (i.e., the selection of a broader bone area around the enthesis), another eight examples are concisely provided in the form of eight Step-Cases (2-9). Nevertheless, **Parts 3 and 4 of the protocol are only visible when selecting Step-Case 1 ("1. OP").** This is to avoid redundancy, given that Parts 3 and 4 are identical for any enthesis and can be fully automated using a Meshlab filter script (as shown in the provided video, below).

5. **IMPORTANT:** In Step 7 (Part 2), Step-Cases 1-5 are accessible via a drop-down menu located below the large "Explanatory Note." However, **Step-Cases 6-9 will only become visible after selecting Step-Case 5 ("5. FDS") and scrolling down to find another drop-down menu. After viewing these additional example cases, you will need to re-select the first Step-Case ("1. OP") from the first drop-down menu to view Parts 3 and 4 of the protocol again.**

Materials

- 3D scanning and processing software: MeshLab (used version: v2023.12)
- 3D surface models of bones with a resolution of at least 300 microns (no texture required)

Before start

Please consider the following recommendations (1 and 2) before applying VERA 2.0:

1. When selecting the muscle or ligament attachment sites (entheses) for analysis in your study, the following must be kept in mind:

- VERA 2.0 is designed to identify projecting surface deformations and irregularities within entheseal areas, broadly referred to in the literature as "entheseal changes". This makes VERA 2.0 particularly effective for detecting such changes in entheses that have the potential to exhibit these alterations at the sample level, even if not present in all individuals. Some key examples of such entheses include the occipital protuberance (cranium), the *masseter* muscle insertion (mandible), the costoclavicular ligament enthesis (clavicle), the deltoid muscle attachment (humerus), the *biceps brachii* enthesis (radius), various key muscle entheses of the hand (e.g., *opponens pollicis*, *extensor pollicis brevis*, *flexor pollicis longus*, *flexor digitorum superficialis*, *opponens digiti minimi*, and the origin sites of the *interossei* muscles), the iliac crest (pelvis), the greater trochanter and supracondylar line (femur), the tibial tuberosity (tibia), the calcaneal tuberosity (calcaneus), and multiple muscle entheses of the pedal metatarsals and phalanges. If there is insufficient bibliographic information on a particular enthesis and its potential changes, it is recommended to visually examine its morphology across your sample and assess whether any consistent irregularities or deformations occur that cannot be exclusively attributed to taphonomy (damage), injury, or inflammatory conditions unrelated to physical activity (e.g., systemic diseases). If an enthesis consistently shows smooth and unaltered surfaces across all individuals in your sample (i.e., no visible entheseal changes), then the traditional "VERA 1.0" approach, which focuses on overall 3D area measurement rather than within-enthesis surface changes, may be more suitable.

- When the aim of the study is to reconstruct habitual muscle or ligament use based on entheseal morphology, the presence of large pathological lesions (e.g., osteophytic or osteolytic) or taphonomic alterations within the enthesis that may likely be unrelated to physical activity can significantly impact the reliability of the measurements. If such alterations are visually identifiable and have a maximum diameter exceeding 4 mm (i.e., the threshold for defining third-level enthesopathies; see criteria in Mariotti et al., 2004), it is hereby recommended to exclude that enthesis from the sample before applying VERA 2.0. Alternatively, if the observer decides to retain the enthesis in the analysis – due to the unique importance of the specimen and/or the suspicion that the potential enthesopathy may be linked to activity-induced biomechanical stress – it is essential to clearly highlight the presence of these alterations in the study's results and interpretations. In cases where such large pathological or taphonomic alterations are within VERA 2.0's "broader selection region" but clearly not within the entheseal footprint region itself, users must ensure that the afflicted areas are manually excluded from the selection (refer to Step-Case 1, Step 11). Similarly, for all remaining lesions below the 4 mm threshold, follow the treatment guidelines provided in Step-Case 1, Step 11.

2. Please note that the delineation process detailed in this protocol is carried out using Meshlab

v.2023.12 (ISTI-CNR, Pisa, Italy). While other software may be used as long as they support the same filtering algorithms, the specific steps outlined here may differ. The predominant portion of the VERA 2.0 workflow (Steps 13 to 23) can become fully automated by using a created Meshlab *mlx* filter script. For convenience, an example filter script is provided for download below, which can also potentially be reused in future studies. If necessary, after loading an existing script in Meshlab, its exact parameters can also be directly adjusted (using the process outlined in the paragraph below). Once the workflow has been applied to the first enthesis of a sample, all subsequent measurements of that enthesis in other individuals can be completed much faster by re-using such a script, potentially taking less than 2 minutes per individual enthesis (as demonstrated in the example delineation video provided further below). This allows for large samples to be analyzed in just a few days.

If observers wish to develop and use their own filter script in Meshlab, they are encouraged to save their filter script after completing the protocol manually for the first enthesis of their sample (Filters → Show Current Filter Script → Save Script). Then, for all other entheses in the sample, as long as the parameters remain unchanged, Steps 13 to 23 can be applied automatically with just a few clicks (Filters → Show Current Filter Script → Open Script → Apply Script). Before running the script each time, it is advisable to quickly double-check that all parameters (and especially the parameter indicated in Step 21, which performs outlier removal based on a specific diameter threshold) are consistent by reviewing them in the "Edit Parameters" tab within the "Show Current Filter Script" window. To ensure consistency, it is strongly recommended to use the same exact script across all 3D models of the sample, avoiding discrepancies in the applied parameters.

Example Video and Reusable Meshlab Filter Script

The video below demonstrates the semi-automated VERA 2.0 approach for analyzing the insertion site of muscle *opponens pollicis* on a human right first metacarpal bone in under 2 minutes. The example delineation video (*mp4*) and the associated Meshlab filter script (*mlx*) can also be found here: **Video and Filter Script Link**. The 3D surface model (*ply* format) was created using an Artec Space Spider structured-light scanner (Artec Inc., Luxembourg) and has a resolution of 100 microns. For background information on the bone depicted in the video, see Karakostis and Hotz (2022).

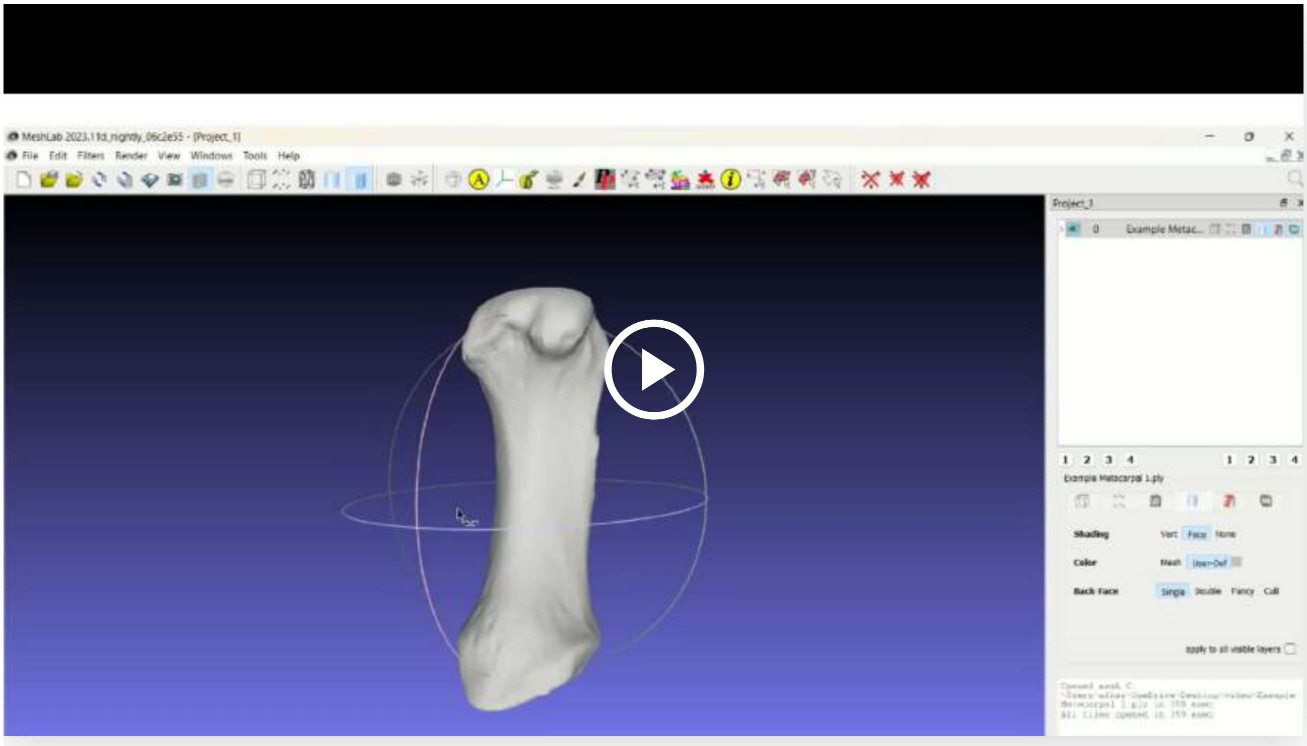

FILE VERA 2.0 filter script.mlx 6KB

Contents:

Part 1 – Technical requirements and recommendations

Part 2 – Selection of a broader bone area around the enthesis (manual component)

Part 3 – Mapping of projecting structures

Part 4 – Delineation and measurement of projecting structures

Part 1 – Technical requirements and recommendations

1 The 3D surface model to be analyzed can be developed using any technology (e.g., laser, structured light, computed tomography, or photogrammetry). The 3D model format used in this protocol is *ply*, but others are also compatible (e.g., *obj* or *stl*). Texture information is not required for VERA

2 Download and use the open-access software Meshlab Version 2023.12 or higher (**https://www.meshlab.net/**), since some steps of this protocol require functions not available in older versions of the software. If another 3D image processing software is used, please ensure that all steps of this protocol are available and that the same (or equivalent) 3D filtering algorithms are employed. Exact specifications on the software's tools and functions can be found in Meshlab's manual and associated references (available at https://www.meshlab.net/#references and https://www.meshlab.net/#support).

3 Confirm that the surface model's resolution is adequate for virtually visualizing entheseal surface changes. Based on trial tests, a minimum resolution of 300 microns is required, though a resolution of 200 microns or lower is strongly recommended for optimal performance.

4 **Importantly, ensure that all 3D surface models in your sample have the same or very similar spatial resolution and mesh structure. Considerable variations in mesh resolution and/or structure, which may occur due to different scanning technologies or selected scanning parameters, can lead to substantial inconsistencies in the identification and measurement of 3D entheseal changes.**

If considerable discrepancies among specimens might be present, it is strongly advisable to standardize the mesh resolution/structure across specimens (surface models) before applying VERA 2.0. This can be done using mesh reconstruction or resampling techniques, such as Meshlab's "Screened Poisson Surface Reconstruction" (with pre-cleaning) or alternatively "Uniform Mesh Resampling" (e.g., with an absolute "Precision" value set to 0.05 for all specimens). The same processing strategy must be consistently applied across all specimens within the sample.

Part 2 – Selection of a broader bone area around the enthesis (manual component)

5 ..

Note

Note: The main example used in this protocol focuses on the insertion site of the muscle *opponens pollicis* on the lateral border of the right first metacarpal bone, relying on a 3D surface model with a resolution of 100 microns (*ply* format), obtained using an Artec Space Spider structured-light scanner (Artec Inc., Luxembourg). The bone itself originates from a modern human female individual of the 19th-century Basel-Spitalfriedhof collection (bioarchaeological remains), previously analyzed in Karakostis and Hotz (2022). All bones depicted in this protocol come from the same collection. All figures provided in this protocol are obtained using the "Save Snapshot" tool of Meshlab. It was explicitly avoided to use direct screenshots depicting the toolbars of the software, to account for future interface changes in future versions of the software that would render such explicit images misleading to the reader. Nevertheless, the text within each Step below pinpoints the exact location of each required function in Meshlab (based on version 2023.12).

Open Meshlab and load the 3D surface model (e.g., either by selecting File ⟶ Import Mesh ⟶ *3D file selection* ⟶ Open, or by "drag & drop"). The 3D surface model is expected to appear (Fig. 1).

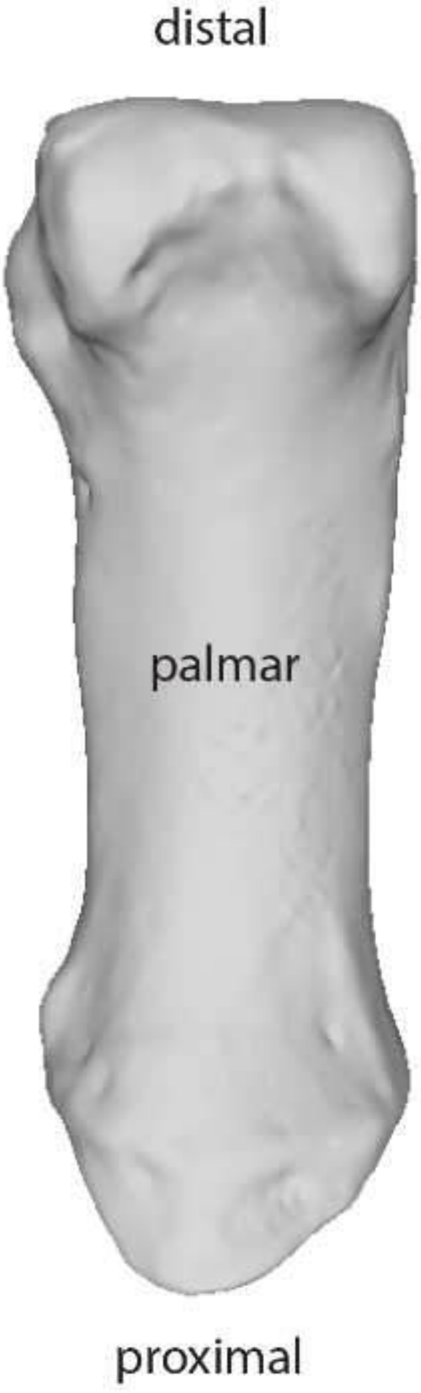

Fig. 1: Snapshot of the example right first metacarpal appearing in Meshlab (or any other 3D image processing software), with anatomical indications. Scale is not respected.

6 If texture is visible on the model (and you do not wish to remove it from the model prior to the analysis), deactivate it by moving your cursor to the right side of Meshlab (Fig. 2) and selecting "Off" next to "Texture Coord" and/or "User-def" next to "Color" (and choosing the grey color, which is the default).

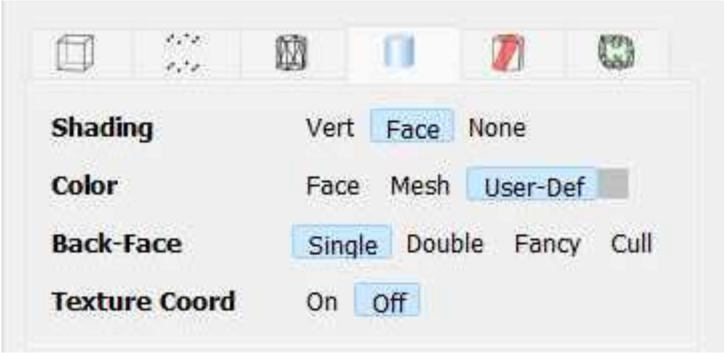

Fig. 2: Snapshot of the panel located on the right side of Meshlab, where it is possible to deactivate 3D surface texture using the indications above.

Note

Explanatory Note on the nine Step-Cases of Part 2 (below):

For applying the semi-automated VERA 2.0 method, the only manual procedure required is the initial selection of a broader, anatomically defined region of the bone surface (Part 2). This region encompasses the enthesis under analysis as well as a significant portion of the surrounding bone area. Once this pre-defined "broader selection region" (BSR) is defined for an enthesis, the rest of the VERA 2.0 workflow (Parts 3 and 4) can be used to automatically detect and measure 3D entheseal changes. Consistency is key: the same BSR must be applied across all specimens for each enthesis under study, to the extent possible (e.g., the same BSR for the *opponens pollicis* insertion on the first metacarpal must be used across specimens). To ensure this consistency, the observer should first establish a specific orientation or viewpoint of the 3D bone model for each enthesis, from which the BSR is marked on the bone surface. This viewpoint is referred to as the "selection perspective" (SP) and has to be the same for each enthesis across specimens. If an SP and BSR for a particular enthesis are not already available in other VERA 2.0 studies, including the nine key example cases of BSR provided below, it is even more critical that the observer defines and clearly depicts them in their study, for future reference.

Within Part 2 (below this Note), I provide detailed recommendations for defining the SP and BSR in the entheses of nine key human muscles or ligaments, in the form of nine separate Step-Cases (1-9). The first six Step-Cases (1-6) focus on the broader area selection procedures for six important entheses of the human hand, chosen because they are among the most frequently analyzed attachment sites using the traditional VERA approach. These examples can also readily serve as a useful reference for defining the BSR of other morphologically similar entheses, such as those of the feet, which share highly comparable anatomical positioning and morphology. The remaining three selective Step-Cases (7-9) address widely studied and important entheses from the shoulder (costoclavicular ligament in the clavicle), arm (deltoid muscle in the humerus), and feet (Achilles tendon in the calcaneus). While these nine example Step-Cases of Part 2 are intended to directly guide future analyses of these specific entheses (and their morphological parallels), their primary purpose is to help future users define a new perspective (SP) and broader region (BSR) for other entheses not covered here. Therefore, I strongly encourage potential VERA 2.0 users to use these examples as a foundation for proposing BSRs for other entheses of the skeleton, particularly in cases where the provided templates may not be readily applicable.

IMPORTANT: As highlighted in the protocol's Guidelines, the entire analytical process (Parts 1 to 4) is described in detail only for the insertion site of muscle *opponens pollicis* (OP) on the right first metacarpal bone. The other eight Case-Studies are only provided for Part 2 (for BSR definition), whereas Parts 3 and 4 of the protocol become only visible when selecting Step-Case 1 ("1. OP"). This is to avoid redundancy given that Parts 3 and 4 are identical for any enthesis and can be fully automated by re-using a filter script (as demonstrated in the delineation example video of the "Before starting" section).

Step-Cases 1-5 can be accessed via the drop-down menu here below, while Step-Cases 6-9 only become visible further below after first selecting Step-Case 5 (i.e., "5. FDS") and then scrolling down to find another drop-down menu. IMPORTANTLY, after observing these example cases, to re-visualize the remaining Parts 3 and 4 of the protocol, you will need to re-select the first Step-case (i.e., "1. OP").

STEP CASE

1. Opponens pollicis (OP) 18 steps

The first Step-Case focuses on the main enthesis example used in this protocol, which is the insertion site of *opponens pollicis* along the lateral border of the right first metacarpal. The instructions in the Step-Case are more detailed than in the others.

7 Using your cursor in Meshlab, rotate the first metacarpal from its initial random position (e.g., the one depicted in Fig. 1) to the specific orientation provided in Figure 3 below. The observer must view the first metacarpal from a fully lateral perspective, with the bone placed in an upright position, the distal end facing upward, and the proximal end downward. The characteristic distal-lateral ridge of the enthesis must be visible to the extent possible (as shown in Fig. 3).

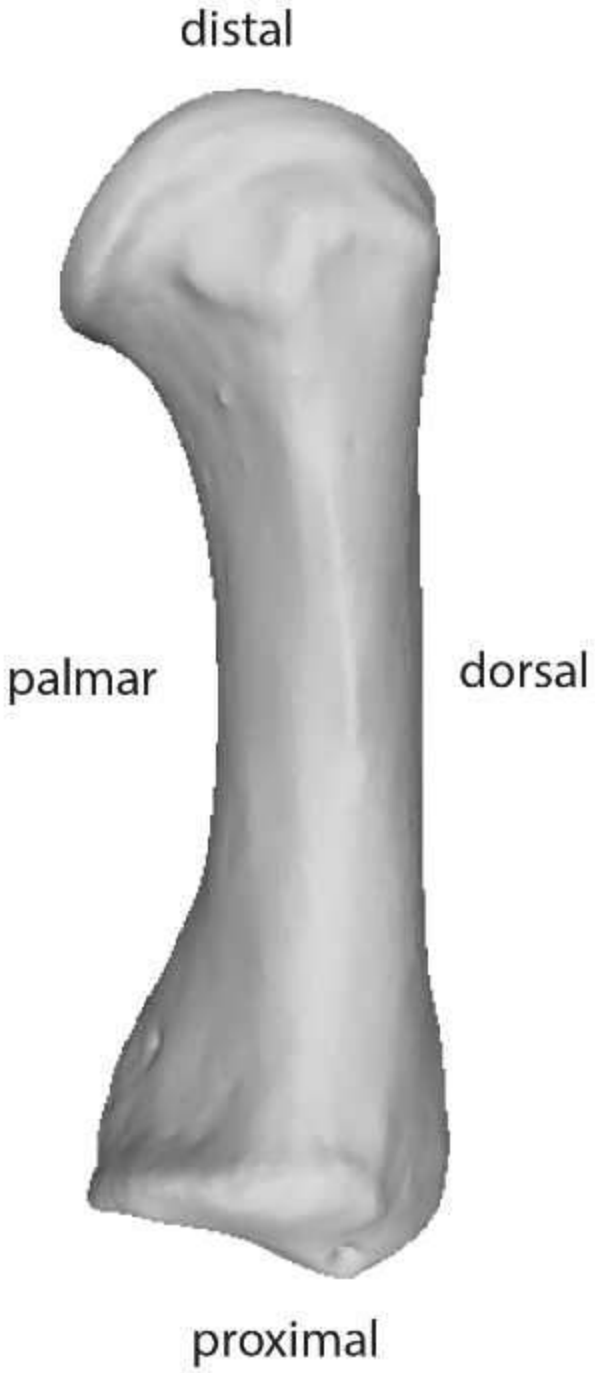

Fig. 3: Selection perspective (SP) for the insertion site of *opponens pollicis* on the right first metacarpal bone. Scale is not respected.

8 Colorize the bone surface using the "Discrete Curvatures" filter (Filters → Colors creation and processing → Discrete curvatures → Apply) and maintaining the default option "Mean" under "Type", to visualize surface curvature in the form of relative projections (in shades of blue) and depressions (shades of red) (Fig. 4). This facilitates the broader selection process indicated below, at Steps 9 to 12.

Note

Note: In case the filter fails to apply due to the presence of manifold edges, then these have to be first removed using the “Repair non Manifold Edges” option (Filters --> Cleaning and Repairing --> Repair non Manifold Edges --> Apply).

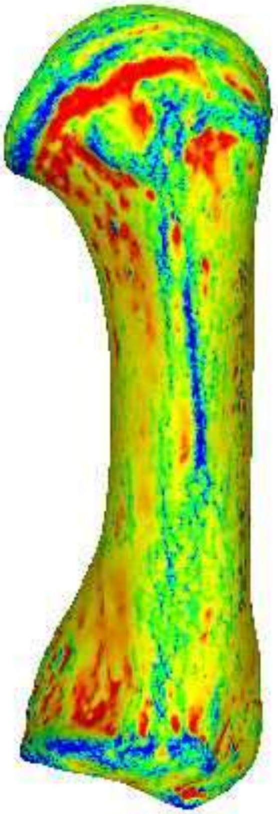

Fig. 4: The selection perspective (SP) after applying the “Discrete Curvatures” filter. Scale is not respected.

9 Select Meshlab's "Z-painting tool" by clicking the appropriate icon with the brush on the right side of Meshlab’s toolbar and then, in the window that pops up, click on the second (lower) brush that is used for manually selecting 3D surface areas. The user can adjust the size and shape of the brush to their convenience (Fig. 5). Areas can be selected by pointing the cursor to the desired location on the model’s surface and pressing the left mouse button. Respectively, areas can be de-selected by pressing the right mouse button.

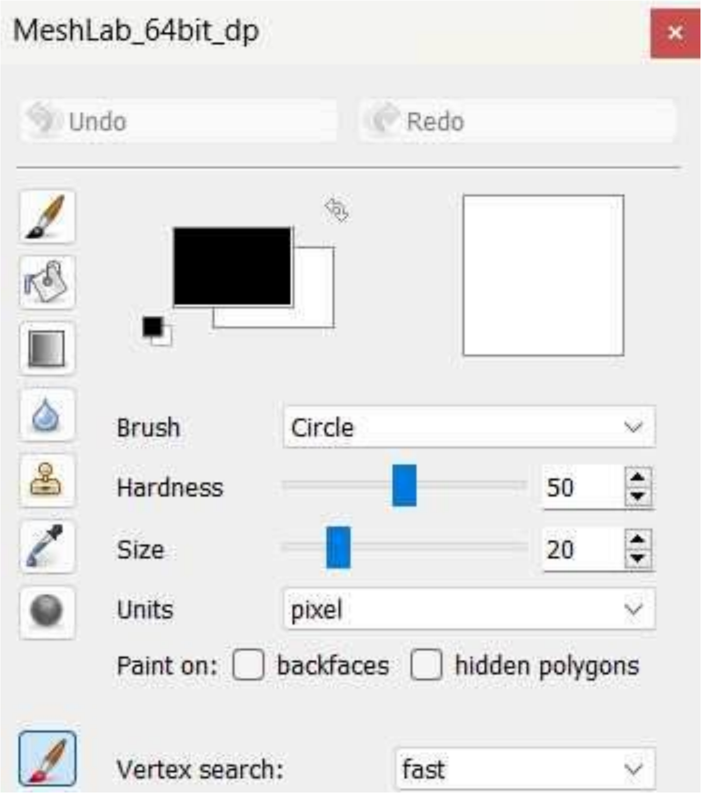

Fig. 5: The window of the Z-painting tool allows the user to adjust the size and shape of the selection brush (also see in the provided example delineation video of the "Before starting" section). The “Discrete Curvatures” filter has been applied in the software Meshlab.

10 When using VERA 2.0 to analyze the insertion site of *opponens pollicis* on the first metacarpal, the user should always select the area indicated in Fig. 6, below. This is defined as the “broader selection region” (BSR) for that enthesis. In particular, it is advised that approximately the distal 4/5ths of the shaft must be selected (not considering the articular surfaces in this estimation), excluding the proximal 1/5th. The latter portion includes the tubercle that accommodates the distinct insertion site of *abductor pollicis longus*, whose distal extent can vary considerably among individuals but is not known to extend beyond 1/5th of the bone. Subsequently, the user must make sure to deselect the articular surface of the metacarpal head, including its rim (typically carrying an elongated blue-colored line, after the “Discrete Curvatures” filter is applied).

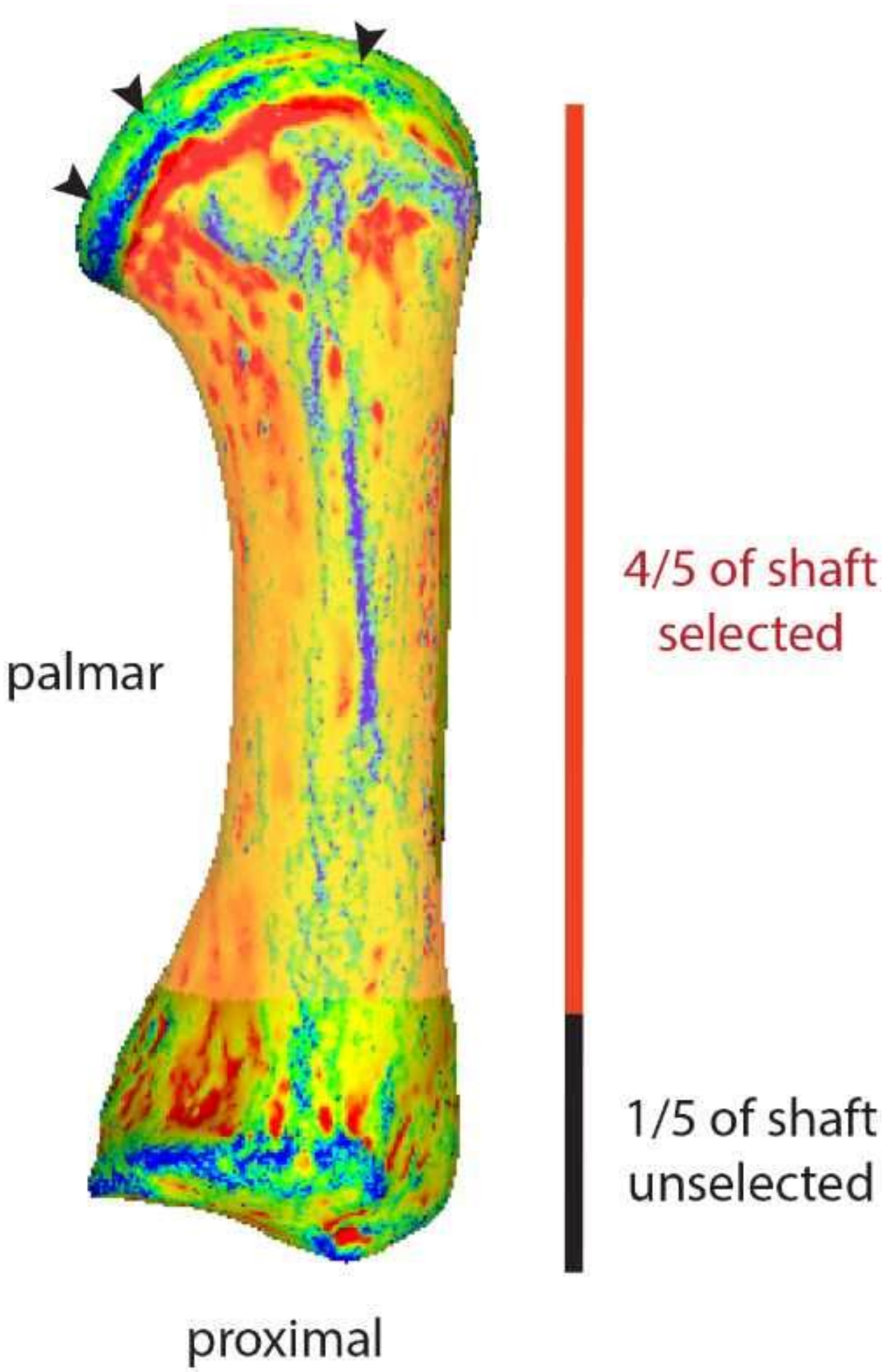

Fig. 6: The area to be selected when analyzing the insertion site of muscle *opponens pollicis* in the first metacarpal (more details in the text above). The user must manually deselect the articular surface and rim of the metacarpal head (indicated here by two black arrows). The "Discrete Curvatures" filter has been applied in the software Meshlab. Scale is not respected.

11 **Additionally, if the selected BSR encompasses clearly identifiable taphonomic alterations and/or pathological lesions (see example in Fig. 7A), these specific regions must also be manually deselected. This also includes alterations within the exact footprint of the tendon**

or ligament, unless the observer explicitly aims to investigate potential links between potential enthesopathy and activity-induced stress in their study (in which case the presence of potential enthesopathies must be highlighted in that study's results and interpretations). If that is the study's aim or intent, then this Step can be intentionally ignored. Otherwise, as explained above (in the "Before starting" section of this protocol), such artifacts must be removed (i.e., manually deselected in the BSR), as they may be associated with taphonomic effects or various pathological reactions unrelated to lifetime muscle or ligament use.

Nevertheless, it should be clarified that when such distinguishable alterations within the BSR are clearly isolated from the main entheseal projections or depressions (thus tending to appear either as blue-colored outlier "islands" or red-colored "pits" after applying the "Discrete Curvatures filter") and their maximum diameter is less than 2 mm (see example in Fig. 7B), they do not necessarily need to be deselected manually, as a later step of the VERA 2.0 workflow (i.e., Step 21) will exclude them automatically. Therefore, such small and isolated structures on the bone surface can be safely ignored, greatly facilitating the manual selection stage required for VERA 2.0. In cases where it is not immediately clear whether an isolated structure surpasses the threshold of 2 mm, the maximum length of a suspected artifact can be quickly measured on the 3D model using the "Measuring Tool" of Meshlab (see examples in Fig. 7). Additionally, the user can easily double-check that such isolated structures did not make it to the final delineation, at the final step of the protocol (i.e., Step 24). If any of them finally did, the observer can choose to select them directly using the marker tool (or other available tools), delete their vertices, and re-measure the remaining delineated area (see steps below).

A

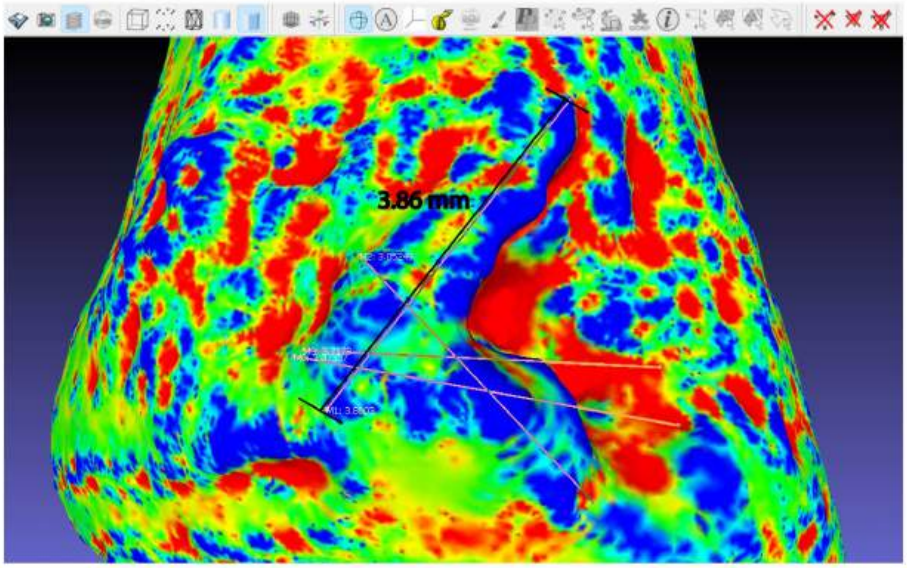

B

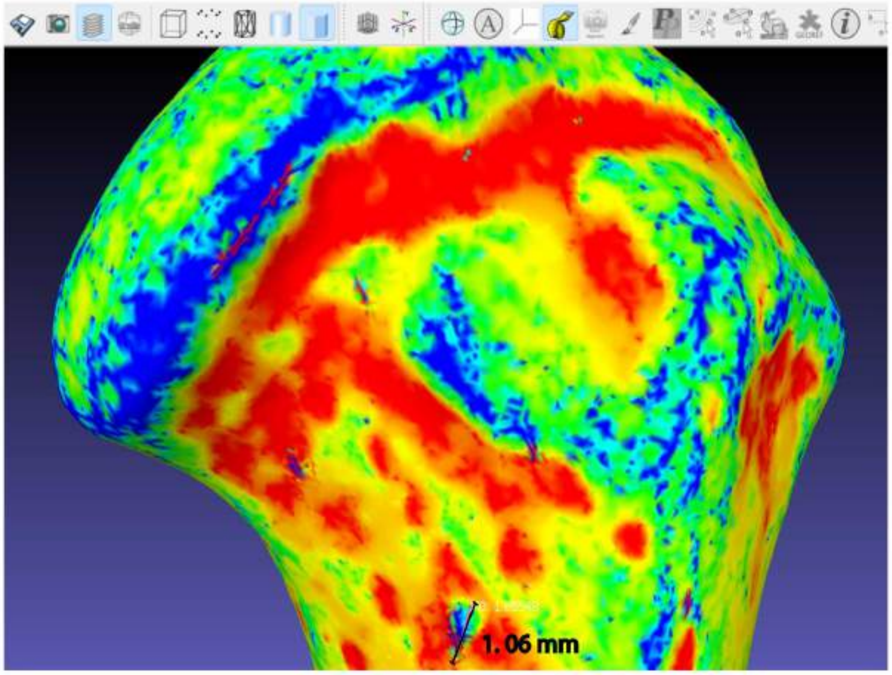

Fig. 7: Example of a relatively large pathological alteration that exceeds 2 mm in maximum diameter (A) and thus needs to be manually deselected before the next steps, and example of a smaller and isolated taphonomic alteration ("blue-colored island") with a maximum diameter of less than 2 mm (B), which does not need to be manually deselected before the next steps. In both cases, Meshlab's "Measuring Tool" was used to measure various diameters on the bone model directly (see black lines and indicated measurements). The "Discrete Curvatures" filter has been applied in the software Meshlab.

12 Finally, the user must ensure that the selection (BSR) did not accidentally

include remote and unrelated aspects of the bone. For this enthesis (*opponens pollicis*), the observer should first rotate the bone model palmarly (Fig. 8A) and ensure that the selection does not surpass the palmar aspect's median (central) axis (indicated here by a black line). Subsequently, the user should view the bone from a dorsal perspective (Fig. 8B) and confirm that the selection does not surpass the dorsal aspect's median (central) axis (indicated here by a black line). Additionally, in case a written label occurs within the selected area (e.g., indicating the specimen label) and this is captured by the 3D scanner, it is advised to manually deselect the labeled selection if possible (as was done in Fig. 8C). Even though the later steps of the automated part of the protocol will usually remove these markings before the final measurement (as long as the lines of the letters are not more than 2 mm in maximum length), it is safer to already manually exclude them at this stage (also see in the provided example delineation video of the "Before starting" section). Finally, the observer is encouraged to additionally observe the bone's metacarpal head from a fully distal perspective, ensuring that the entire articular surface is clearly visible (to the extent possible). This allows for confirmation that no part of the distinguishable articular surface's rim was accidentally selected (as demonstrated in the provided video).

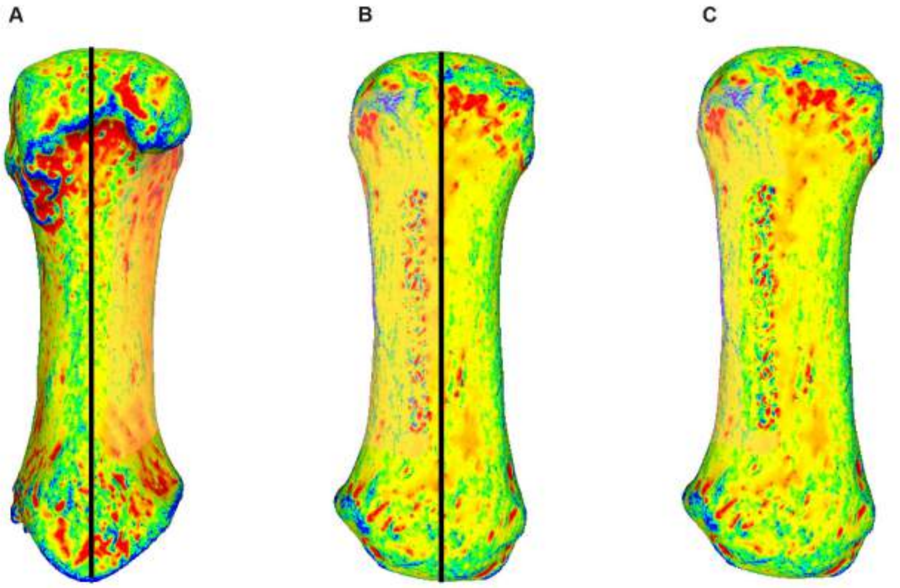

Fig. 8: Palmar (A) and dorsal (B) aspects of the bone with a vertical black line indicating the median limit of the selection. Areas accidentally selected that surpass this limit must be deselected before the next steps. Panel C shows the deselection of a written specimen label on the bone surface, which is recommended in cases where the ink may have considerably affected the surface curvature. Scale is not respected.

Part 3 – Mapping of projecting structures

13

Note

NOTE: The remaining VERA 2.0 steps are consistent across all entheses and analyses. These can therefore be completely automated by relying on a stored Meshlab filter script (*mlx*), which can be applied after selecting the BSR for each enthesis (see example video and script provided in the "Before starting" section). For illustration, these steps are demonstrated here using the insertion site of the *opponens pollicis* muscle on the right first metacarpal. After completing all subsequent steps required for entheseal measurement once (from Step 13 to Step 23), it is recommended that the observer saves the resulting filter script file in Meshlab. This script can then be re-loaded and reused for all other specimens and entheses in the sample, following the selection of the BSR in each case.

Select the vertices corresponding to your BSR in Meshlab (Filters ⟶ Selection ⟶ Select Vertices from Faces) (Fig. 18). Subsequently, apply Laplacian smoothing on the selected area, (Filters ⟶
Smoothing, Fairing and Deformation ⟶ Laplacian Smooth ⟶ Apply), maintaining Meshlab's default number of smoothing steps (3).

Importantly, the smoothing step may be omitted in cases where the 3D model's surface appears to be already relatively smooth (e.g., due to low scanning resolution and/or other parameters) and further smoothing seems to clearly reduce relevant surface information (i.e., parts of distinctive entheseal changes unrelated to pathology or taphonomy). When in reasonable doubt, simply running VERA 2.0 both with and without the smoothing step (i.e., re-using the same *mlx* filter script after editing it in Meshlab to remove the initial smoothing step) is recommended. The resulting area measurement datasets from both approaches (with and without smoothing) can then be analyzed and statistically compared. For each analysis, the same smoothing parameters/strategy must be applied across the sample under study, to ensure consistency across specimens.

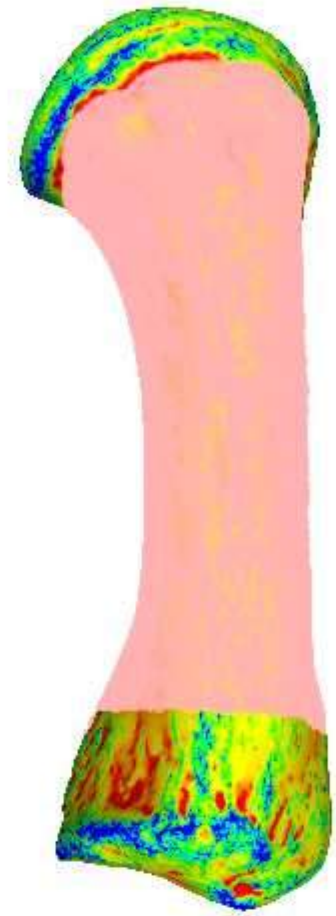

Fig. 18: Snapshot of the right first metacarpal depicting the selected vertices of the broader selection region (BSR) for the example enthesis of *opponens pollicis* after applying Laplacian smoothing.

Note

Note: Laplacian smoothing averages the position of each vertex with its neighbors, preserving broader structures while reducing smaller, sharper anomalies. Bone surface scans may often contain small, sharp projections from noise, artifacts, or various surface irregularities that aren't necessarily meaningful anatomical variations. Smoothing helps highlight continuous, representative projections on the bone surface by reducing these artifacts.

14 Select the bone areas surrounding the BSR itself by inverting the current selection (Filters ⟶ Selection ⟶ Invert Selection) (Fig. 19).

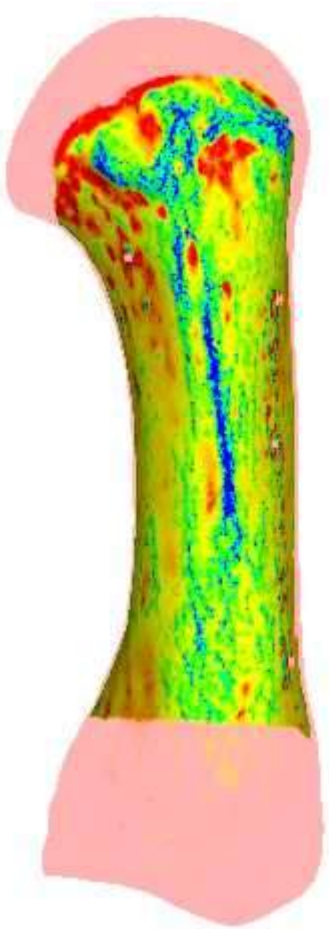

Fig. 19: Snapshot of the right first metacarpal and its broader selection region (BSR) after inverting the selection of vertices. Scale is not respected.

15 Crop the selected bone regions surrounding the BSR, using the "Delete Selected Vertices" tool located on the right side of Meshlab's toolbar (Fig. 20).

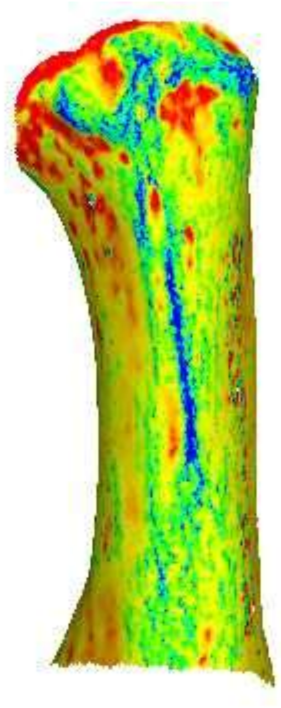

Fig. 20: Snapshot of the broader selection region (BSR) after cropping the selected surrounding bone areas. Scale is not respected.

16 To enhance the distinction of all smoothly projecting areas on the bone surface (i.e., blue-colored structures in the figure), apply colorization to the BSR surface based on computed principal curvature directions (Filters → Color Creation and Processing → Compute Curvature Principal Directions). In the pop-up panel, use the software's default method "Quadric Fitting" and set the quality to "Min Curvature." This approach will colorize each vertex of the BSR according to the minimum observed curvature, which is ideal for capturing more subtle surface variations (Fig. 21). In case the filter fails to apply due to the presence of manifold edges, then these have to be first removed using the "Repair non Manifold Edges" option (Filters ⟶ Cleaning and Repairing ⟶ Repair non Manifold Edges ⟶ Apply).

Note

Note: Using the minimum curvature criterion ("Min Curvature") at this stage highlights a wider range of less pronounced features of surface morphology, providing a more extensive representation of projecting areas compared to "Max Curvature," which focuses on the sharpest and most localized features, often resulting in fewer blue-colored areas indicated after applying the filter. The use of "Mean Curvature", on the other hand, averages both maximum and minimum curvatures, which can potentially reduce the contrast between sharp and subtle features, making it likely less effective for distinguishing smoothly projecting areas.

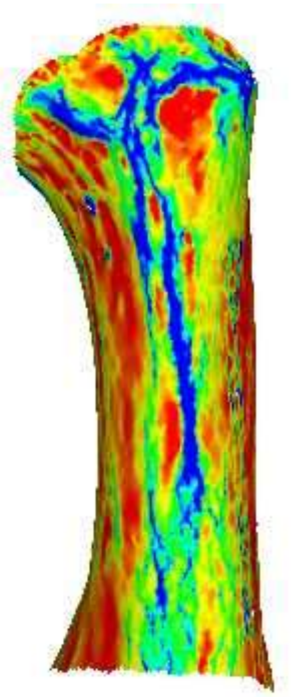

Fig. 21: Snapshot of the broader selection region (BSR) after colorizing the bone based on principal curvature directions. The colorization relied on the "Quadric Fitting" method and the minimum curvature ("Min Curvature") quality setting. Projecting regions are highlighted in blue shades.

17 To standardize all shades of blue in the BSR, equalize the color histogram to ensure uniform blue shades (Filters → Color Creation and Processing → Equalize Vertex Color → Apply) (Fig. 22).

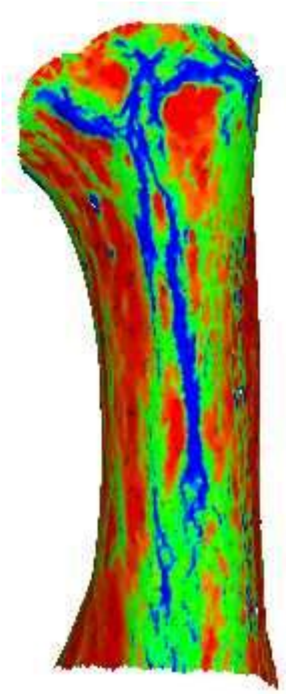

Fig. 22: Snapshot of the broader selection region (BSR) after colorizing the bone based on principal curvature directions (Fig. 21) and applying histogram equalization to standardize the blue shades. Scale is not respected.

Part 4 – Delineation and measurement of projecting structures

18 Select all projecting areas in the BSR, which should appear in blue after the previous colorization process (Fig. 23). Navigate to "Filters → Selection → Select Faces by Color". In the pop-up window, choose the blue color (#0000ff) and set "Variation from Blue or Value" to the maximum (1.0). Leave the remaining settings at their default values and click Apply.

Note

Note: Setting the "Variation from Blue" to 1.0 ensures that all shades of blue, including any lighter blue that may remain after equalization, are included in the selection.

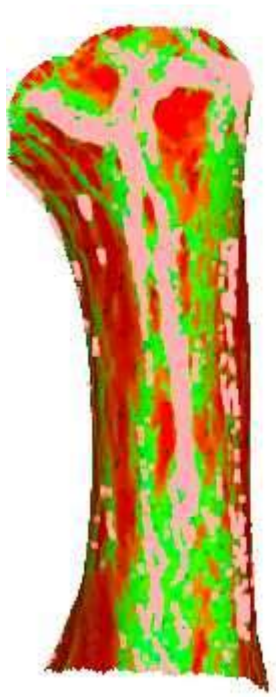

Fig. 23: Snapshot of the broader selection region (BSR) showing the selected blue regions (highlighted) after applying the colorization process described in the previous steps. Scale is not respected.

19 Invert the selection (as previously described in Step 14) to select all areas of the BSR except for the previously selected blue regions (Filters → Selection → Invert Selection) (Fig. 24).

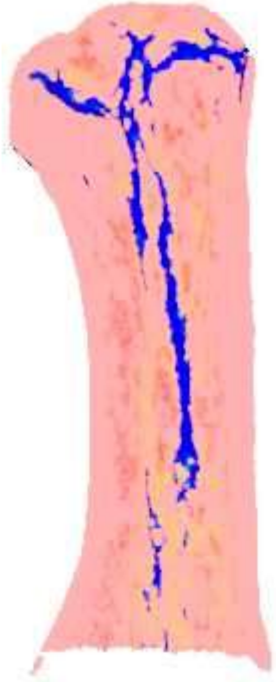

Fig. 24: Snapshot of the broader selection region (BSR) after inverting the selection to highlight all areas except the blue regions previously selected. Scale is not respected.

20 Crop the selected regions surrounding the blue-colored projecting areas, using the “Delete Selected Vertices” tool located on the right side of Meshlab’s toolbar (Fig. 25).

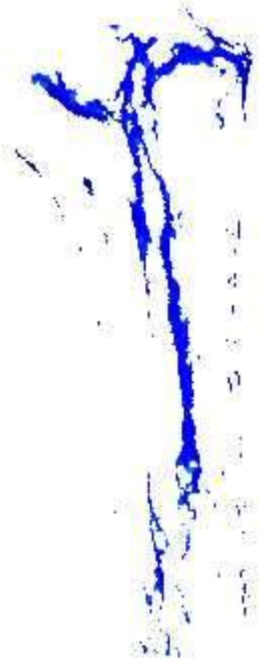

Fig. 25: Snapshot of the remaining, blue-colored regions after cropping the surrounding areas that were selected previously. Scale is not respected.

21 In the remaining surface (now entirely blue), automatically detect and remove small and unconnected bone projections ("outliers" or "islands"), as these cannot typically be safely associated with the main entheseal changes observed (Filters → Cleaning and Repairing → Remove Isolated Pieces - wrt Diameter → Apply). In the pop-up window, set the diameter threshold to 2 mm and then click Apply (Fig. 26). This will remove all isolated connected components (outliers) whose diameter is below that threshold.

Note that the percentage (%) automatically indicated next to that value (2 mm) tended to be around 5% for the example human specimens analyzed for developing this protocol and its associated validation paper.

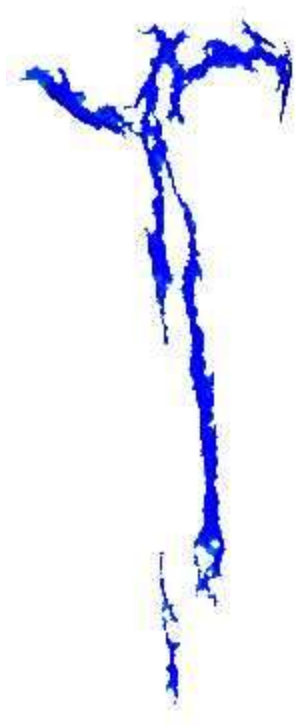

Fig. 26: Snapshot of the remaining, blue-colored regions after automatically removing isolated pieces of 2 mm or less. Scale is not respected.

Note

Note: If setting the threshold to 2 mm removes all blue areas in the model (resulting in "0" areas) or reduces structures that are clearly critical for the analysis (i.e., visually identifiable entheseal changes unrelated to pathology or taphonomy), likely due to the small size and/ or less smooth morphology of the 3D model, one option is to simply represent this particular enthesis in your dataset with a "0" area measurement (or any other actual outcome). In this case, the loss of potentially relevant entheseal information following that step has to be acknowledged and discussed in the study.

Alternatively, you may choose to repeat the entire delineation process (i.e., re-run the script) with the outlier removal threshold set to 1 mm instead of 2 mm (note: this parameter/step can also be directly edited and saved in a loaded Meshlab filter *mlx* script). Then, if the threshold of 1 mm also reduces critical entheseal features or even the entire blue area, which can indeed occur if the enthesis is very small and/or smooth (e.g., as in some entheses of small laboratory animals), you may also choose to skip this step (Step 21) altogether. In some cases, the latter is the best strategy for avoiding to remove clearly relevant entheseal information. Nevertheless, if you decide to skip this step or change the threshold for one specimen, then ensure that this decision is applied consistently across all specimens of the same enthesis in your sample.

22 Select all remaining regions (Filters ⟶ Selection ⟶ Select All) (Fig. 27).

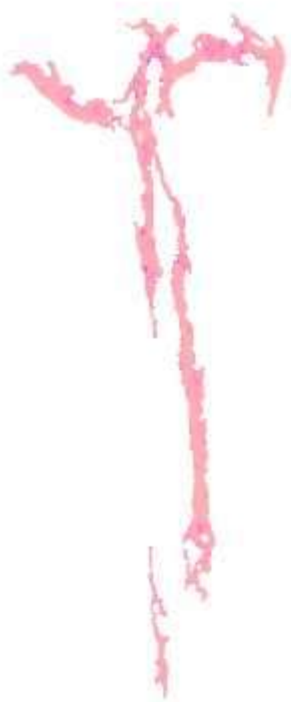

Fig. 27: Snapshot of all remaining blue regions selected. Scale is not respected.

23 Measure the surface area of the final selected blue regions in square mm (provided the model is calibrated accordingly) by navigating to "Filters ⟶ Quality Measure and Computations ⟶ Compute Area/Perimeter of Selection". The "Selection Surface Area" measurement will be displayed in the window at the bottom right corner of Meshlab (Fig. 28). The exact location of the measurement indication is also highlighted in the example delineation video provided in the "Before starting" section.

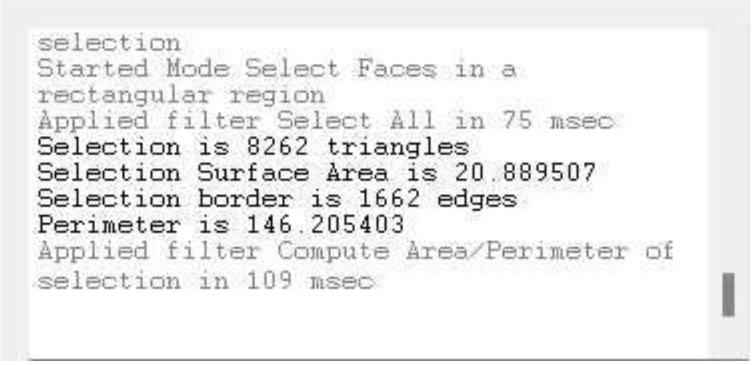

Fig. 28: Display of the surface area measurement in the bottom right corner of the Meshlab window.

24 Optionally, you may project the delineated entheseal changes onto the bone 3D model of the bone by importing the original 3D model file (e.g., the *ply* file of the first metacarpal) into Meshlab. To overlay this model, you may "drag and drop" the file into the same workspace where the processed model is displayed, by holding down the left mouse button during the action. This overlay process, as illustrated in Fig. 29 as well as the example delineation video provided in the "Before starting" section, allows you to visualize the selected regions on top of the bone model. It provides an opportunity for final verification to ensure that the workflow was executed correctly and to check for any potentially irrelevant areas still selected (e.g., areas outside the defined BSR or regions with clear taphonomic or pathological damage). If additional adjustments are needed, such as eliminating erroneously selected areas using Meshlab's selection tools, document the decision and reasoning clearly in your study's Methods section (optimally using illustrations). Eight additional examples of this process are illustrated below in Fig. 30, corresponding to the eight example cases of BSR selection discussed in Part 2 of this protocol (Case-Steps 2-9).

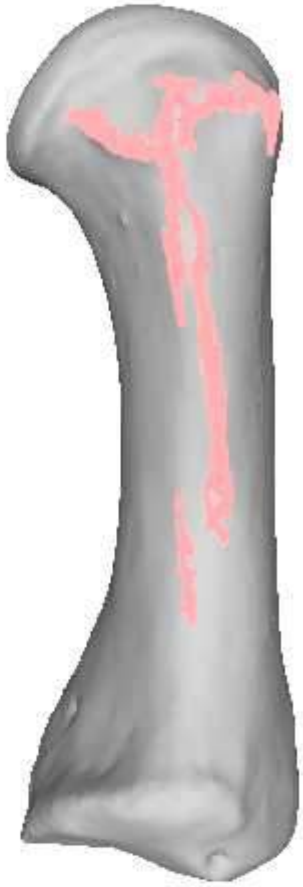

Fig. 29: Optional overlay of the selected regions (3D entheseal changes) onto the original *ply* model of the first metacarpal for final verification. Scale is not respected.

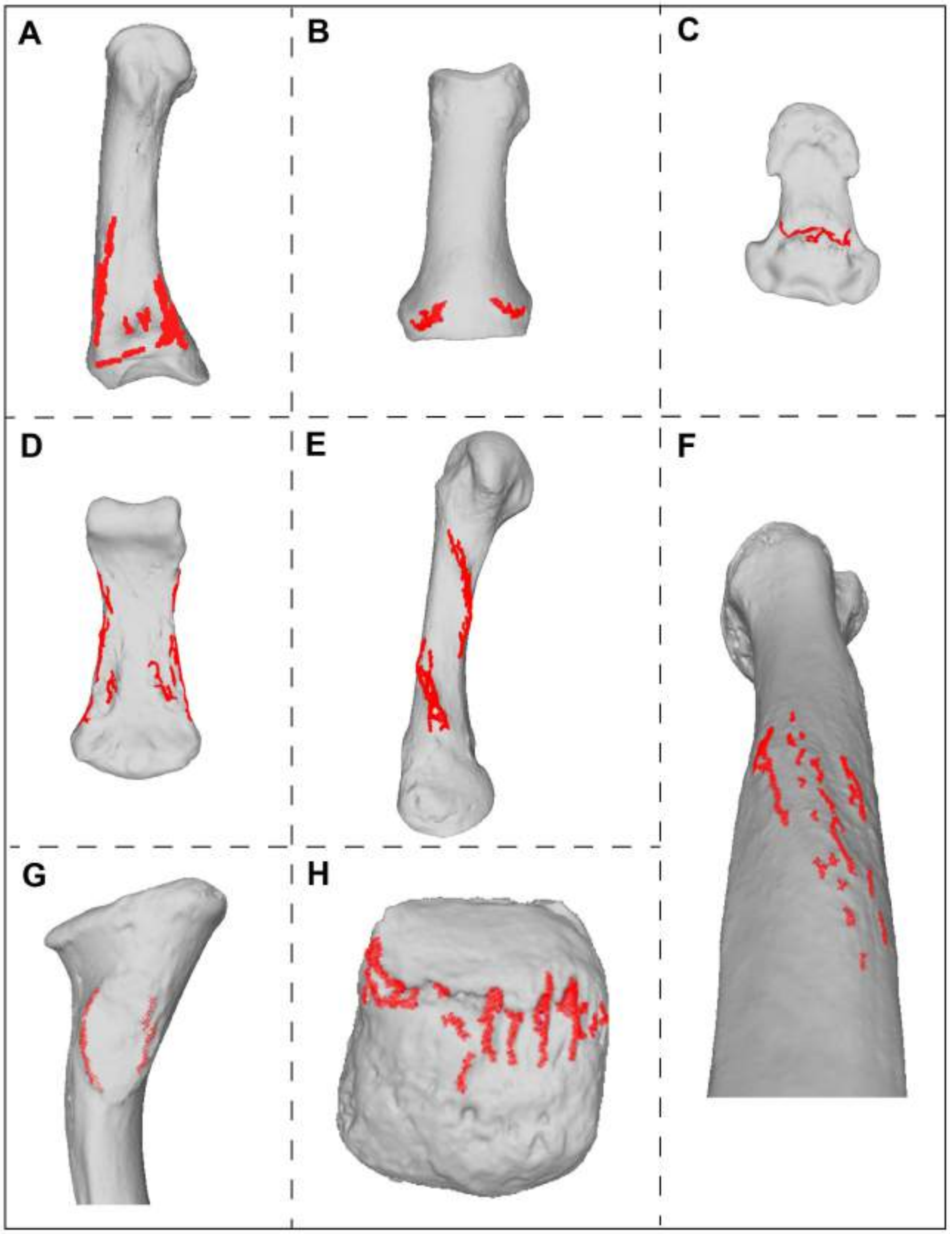

Fig. 30: Additional examples of the optional overlay step, showing the selected and measured entheseal changes on the original bone 3D model for final verification. These examples correspond to the eight case studies and delineations discussed above in Part 2 (Case-Steps 2-9). In particular, the examples include the attachment sites of the following tissues: the second dorsal *interosseus* on the first metacarpal (A), *extensor pollicis brevis* on the first proximal hand phalanx (B), *flexor pollicis longus* on the first distal hand phalanx (C), third *flexor digitorum superficialis* on the third intermediate hand phalanx (D), *opponens digiti minimi* on the fifth metacarpal (E), deltoid on the humerus (F), costoclavicular ligament on the clavicle (G), and Achilles tendon on the calcaneus (H). Scale is not respected across panels.

STEP CASE

2. First dorsal interosseus (DI1) 1 step

For the origin site of the first dorsal *interosseus* (first metacarpal):

Part 2 – Selection of broader bone area around the enthesis (manual steps)

7 **Case 2 Steps 7-12 (summary):** In this example enthesis, the 3D surface model of the first metacarpal has to be manually rotated to obtain the SP shown in Fig. 9A. In particular, only the medial aspect of the palmar diaphysis must be clearly visible at the center of the viewpoint while the bone is placed in an upright position (distal up). As shown in Fig. 9A, after applying the "Discrete Curvatures" filter, the marker tool must be used to select a BSR that begins from the visible midshaft of the bone (distally) and expands proximally until the height of the most distal point of the proximal articular surface's rim (which is indicated in the figure by the three lower black arrows). The rim of the proximal articular surface itself (typically represented by lines of blue, after applying the above filter) must not be included in the selection. Subsequently, the 3D model must be rotated twice to view its palmar-lateral and fully dorsal aspects (see Fig. 9B, left and right) and ensure that these two entire regions/ aspects are entirely removed from the selection (with the right mouse button). This additional step leads to the final BSR highlighted in Fig. 9A, which covers most of the proximal half of the bone's palmar-medial shaft, excluding the articular surface's rim, the areas underneath it, and a very thin visible part along the bone's palmar and dorsal aspects (indicated by the left black arrow).

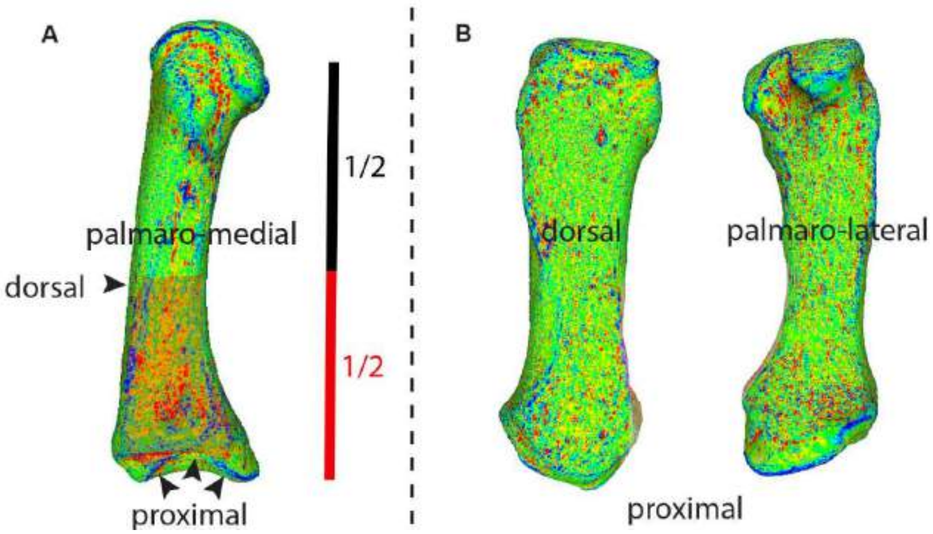

Fig. 9: Selection perspective (SP) and highlighted broader selection region (BSR) for the origin attachment site of the first dorsal interosseus on the right first metacarpal bone (A). Panel B shows the two additional bone aspects used to deselect all dorsal and palmar-lateral regions. The "Discrete Curvatures" filter has been applied in the software Meshlab. Scale is not respected.

STEP CASE

3. Extensor pollicis brevis (EPB) 1 step

For the insertion site of the *extensor pollicis brevis* (first proximal hand phalanx), which can also act as a basis for the insertion site of the *extensor hallucis brevis* (first proximal foot phalanx):

7 **Case 3 Steps 7-12 (summary):** For this enthesis, the 3D model of the proximal phalanx has to be viewed from a fully dorsal perspective (i.e., hereby defined as a viewpoint from which the dorsal basal dimension of the bone appears to be the widest), as shown in Fig. 10A. This is often a view in which the thumb's phalangeal head tends to often appear slightly tilted (as shown in the example of Fig. 10A). After applying the "Discrete Curvatures" filter (maintaining the above SP), the entire proximal third of the visible dorsal aspect's shaft (not considering the two articular surfaces when estimating total bone length) must be selected using the marker tool, excluding the rim of the articular surface (the lowest visible border of the SP) that is typically distinguishable by the presence of blue lines (as indicated by the two lower black arrows in Fig. 10A). Subsequently, the bone has to be rotated twice, palmar-laterally and palmar-medially (exactly as shown in Fig. 10B), and the user must ensure that the main bodies of the two tubercles (which correspond to other entheses of the thenar musculature, unrelated with the *extensor pollicis brevis* muscle) are removed from the selection (using the right mouse button). Consequently, as shown in Fig. 10A, the final selection before analysis will not include the two tubercles highlighted by the two upper

black arrows in Fig. 10A, atensure the corners of the BSR.

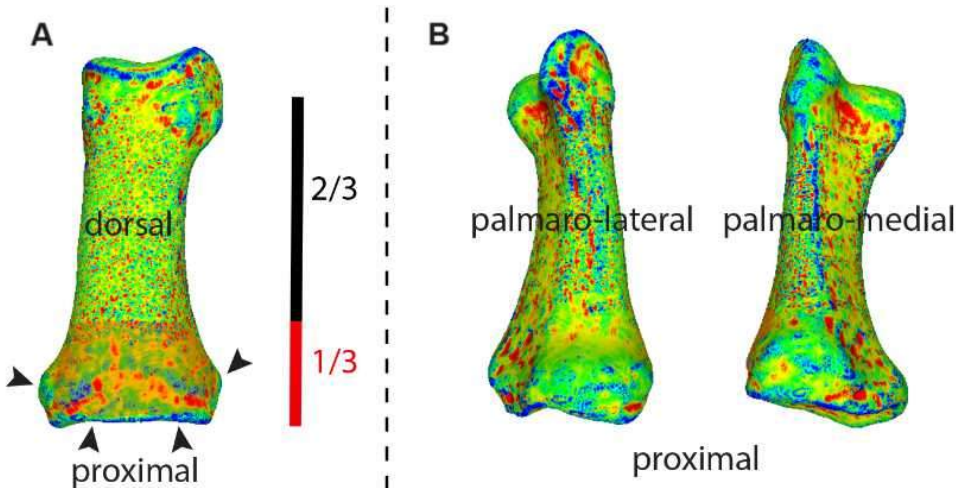

Fig. 10: Selection perspective (SP) and highlighted broader selection region (BSR) for the insertion attachment site of the *extensor pollicis brevis* on the right first proximal hand phalanx (A). Panel B shows the additional aspects used to ensure that no areas were accidentally selected palmar-laterally and palmar-medially. The "Discrete Curvatures" filter has been applied in the software Meshlab. Scale is not respected.

STEP CASE

4. Flexor pollicis longus (FPL) 1 step

For the insertion site of the *flexor pollicis longus* (first distal hand phalanx), which can act as a basis for the insertion site of the *flexor hallucis longus* (first distal foot phalanx):

7 **Case 4 Steps 7-12 (summary):** For this enthesis, the 3D bone model must only be viewed from a full palmar perspective, from which the palmar mediolateral midshaft width appears to be the longest (as shown in Fig. 11). As with the other example entheses, the bone has to be placed in an upright position, with the distal apical tuft facing upward. Here, the roughly rectangular BSR must begin from the level of the apical tuft (i.e., a horizontal line linking the two most proximal points of the apical tuft, indicated by the upper black arrow in Fig. 11) and expand proximally until another oblique straight line that links the two most proximal points of the arch-shaped ridge, where the flexor pollicis longus is known to attach. In the example of Fig. 11, the two ends defining this line are indicated by the lowest two black arrows. Finally, to ensure that no medial and lateral borders of the palmar bone shaft are accidentally selected (even though they tend to be slightly visible from a palmar perspective), the observer must deselect an extremely thin line along the medial and the lateral borders of the selection (indicated by the remaining two black arrows in

Fig. 11, pointing medially and laterally). It is important that the entire arch-shaped ridge (indicated by the lowest two black arrows) is finally included in the BSR.

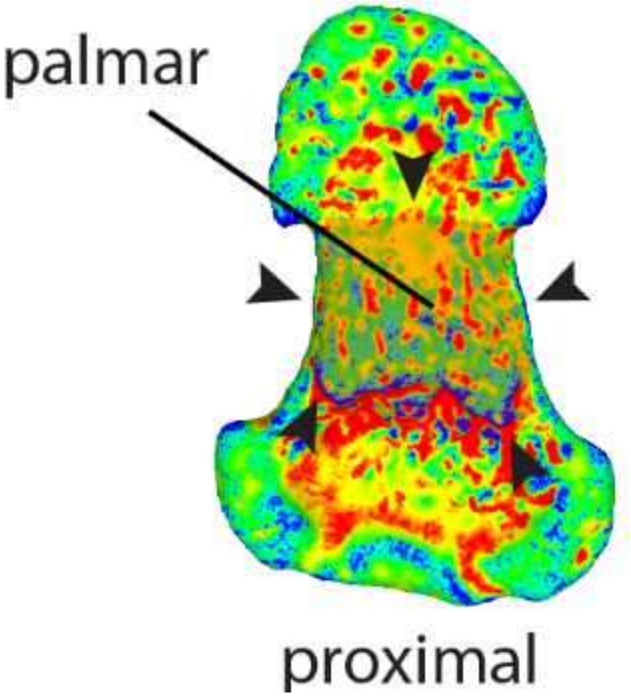

Fig. 11: Selection perspective (SP) and broader selection region (BSR) for the insertion attachment site of the *flexor pollicis longus* on the right first distal hand phalanx (thumb). The "Discrete Curvatures" filter has been applied in the software Meshlab. Scale is not respected.

STEP CASE

5. Flexor digitorum superficialis (FDS) 2 steps

For the insertion site of the *flexor digitorum superficialis* (third intermediate hand phalanx), which can act as a template for all other flexor entheses on intermediate hand phalanges:

7 **Case 5 Steps 7-12 (summary):** This enthesis is associated with two palmar ridges (one medial and one lateral) on the shaft of intermediate phalanges (Fig. 12A). The 3D bone model must be viewed from a full palmar perspective (i.e., the palmar mediolateral width must be the longest possible), while the bone is placed in an upright position with its distal end facing upward (Fig. 12A). After enabling the "Discrete Curvatures" filter, the presence of the two flexor ridges is highlighted by a mixture of blue and green color shades on the visibly elevated areas. The entire spread of each of these two elevated regions must be included in the BSR, spanning from the neck of the phalanx distally

(indicated by the two upper black arrows in both panels of Fig. 12) until the level of the two distinctive basal projections proximally (indicated in Fig. 12 by the lower two black arrows), which are adjacent to the articular surface rim. It is advised that these two basal projections are not included in the selection. Subsequently, the observer must rotate the bone twice (first palmar-medially and then palmar-laterally, as shown in the two images of Fig. 12B), to ensure that no considerable areas were accidentally selected dorsally to each distinguishable ridge (i.e., the elevated elongated surface). In each of the two images of panel B, the approximate dorsal limits of the two ridges are indicated by a black arrow.

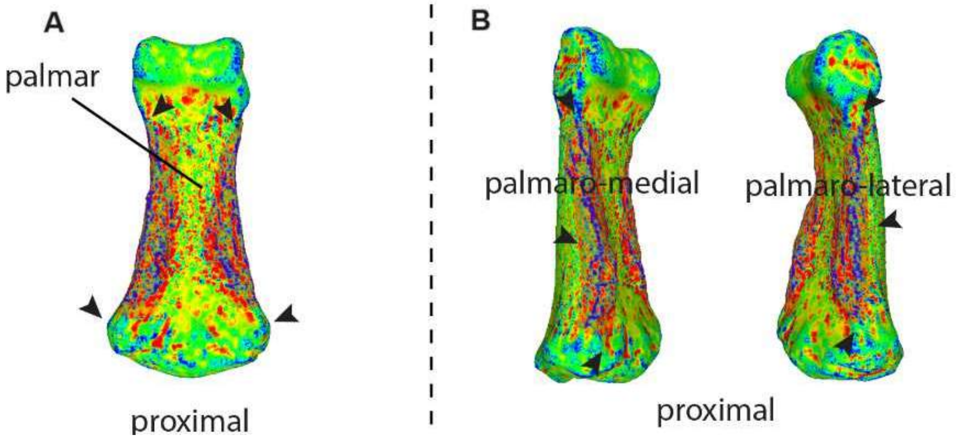

Fig. 12: Selection perspective (SP) and broader selection region (BSR) for the insertion attachment site of the third *flexor digitorum superficialis* on the right third intermediate hand phalanx (A). Panel B shows the additional aspects used to reassure that no areas were accidentally selected palmar-medially and palmar-laterally (B). The "Discrete Curvatures" filter has been applied in the software Meshlab. Scale is not respected.

STEP CASE

Opponens digiti minimi (ODM) 1 step

For the insertion site of the *opponens digiti minimi* (fifth metacarpal), which can act as a basis for all origin entheses of the interossei muscles in metacarpals and metatarsals 2-5:

7.1

Note

Note: This fibrous enthesis covers an extensive portion of the fifth metacarpal's medial shaft and, based on previous VERA 1.0 studies on extensively documented human skeletons, its variation shows clear associations with habitual muscle use and long-term physical activity (Karakostis and Hotz, 2022). However, before analyzing this enthesis using the VERA 2.0 protocol, an additional preliminary consideration is required because many individuals do not present any distinctive entheseal changes in this bone region (for example, see the left image of Fig. 13), while it is quite common for subtle surface alterations of variable etiologies (i.e., numerous small projections and depressions) to occur across metacarpal shafts (for example, in the right image of Fig. 13, after application of the “Discrete curvatures” filter, see the multiple blue-colored isolated structures across the bone). Consequently, directly selecting a pre-defined large BSR for this enthesis in the absence of any clear and sizeable entheseal changes may lead to inflated 3D area measurements because the subsequent VERA 2.0 measuring flowchart (Step 21) might fail to identify such isolated small alterations as “outliers” and thus eliminate them from the final entheseal measurement.

Therefore, before selecting the BSR described below for that enthesis, the user should first directly observe the medial shaft of the fifth metacarpal without applying any filters at all (e.g., left image of Fig. 13), to visually evaluate if any identifiable entheseal changes visually occur across the bone's medial shaft. If they do not (as in Fig. 13, left image), the observer is advised to stop the process here and simply indicate a “0” area measurement for that specimen (accompanied by an added clarification on what this signifies). In fact, the absence of visible entheseal changes in some individuals of a comparative sample (but not in others) can be highly informative when reconstructing physical activity based on bone morphology. However, in case a potential entheseal change is suspected, the observer can additionally apply the “Discrete Curvatures” filter (as in Fig. 13, right image) and further observe if any of the suspected entheseal changes (blue-colored projections) are sizeable (i.e., at least 5 mm in maximum length) and do not show typical characteristics of taphonomic and/or pathological nature (e.g., the presence of several small irregular or rounded “islands” or pits on the surface, like for example the multiple blue-colored artifacts indicated by black arrows in the right image of Fig. 13). If one or more of these distinctive elongated structures measuring at least 5 mm are present, such as the two shown in Figure 14, the observer should proceed with the steps outlined below. Additional examples of such cases can be found in the fourth figure of the study by Karakostis and Hotz (2022).

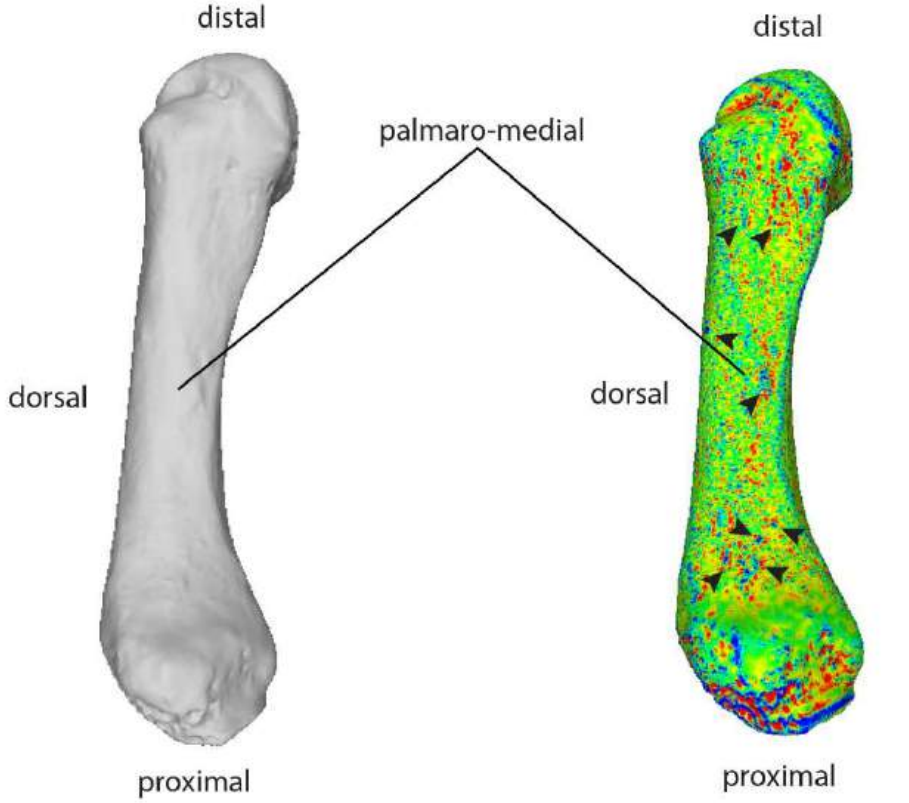

Fig. 13: The selection perspective (SP) of a right fifth metacarpal that lacks considerable entheseal changes (based on the criteria explained in the above Note). The left image shows the bone without any filters applied, while the right image shows the same aspect after applying the filter “Discrete Curvatures”. The black arrows indicate examples of small isolated surface alterations (appearing in shades of blue) which are likely of a taphonomical nature and do not meet the above size criteria recommended for measurement (at least 5 mm in maximum length). Scale is not respected.

Case 6 Steps 7-12 (summary): For this enthesis, the 3D bone model must be viewed from a palmar-medial perspective (i.e., where the maximum width of the palmar-medial midshaft width appears to be the longest possible), as shown in Fig. 14A. As in the other examples/cases above, the bone must be placed again in an upright position, with the distal end (head) facing upward. The palmar median crest of the bone separating the palmar shaft into two components (medial and lateral) must not be directly visible in the SP (to the degree that this is possible), as indicated within the images of Fig. 14. The BSR must approximately include the middle 2/4ths of the total shaft length (not considering the articular surfaces from the estimation), thus excluding the large proximal tubercle acting as the insertion site of extensor carpi ulnaris as well as the neck of the metacarpal head (distally). Subsequently, as shown in

Fig. 14A, the user must deselect a very thin line along the dorsal border (highlighted by the three left black arrows of Fig. 14A), to ensure that no dorsal aspects of the bone are selected. Optionally, rotating the bone dorsally (as it was previously done for the first metacarpal in Fig. 8) can further fully confirm that no dorsal aspects were accidentally selected. Finally, in case any small traces of the palmar median ridge are still visible within the BSR (see black arrow with text indication in Figs. 14A and 14B), these must be manually deselected too. Finally, the three lower black arrows in Fig. 14B show examples of minor "blue-colored" alterations with a diameter of less than 2 mm, which will be automatically excluded in the next steps of VERA 2.0 (Step 21), as long as any sizeable entheseal structures are also present in the first place (as explained in the above **Note**).

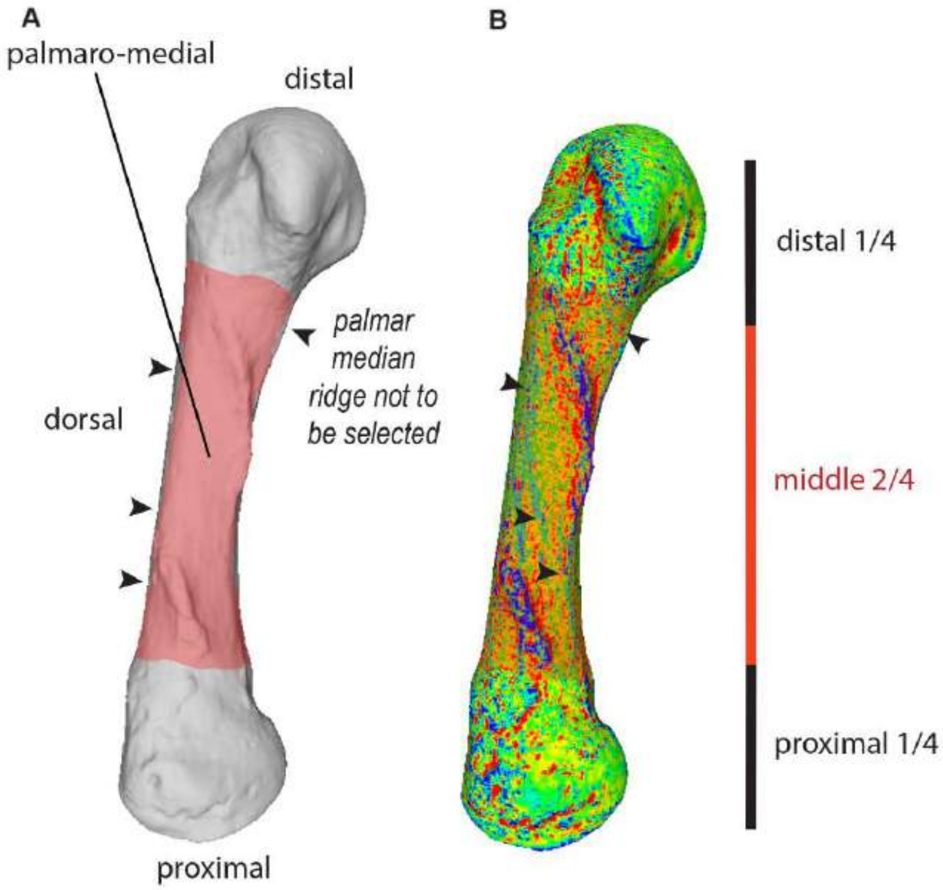

Fig. 14: Selection perspective (SP) and broader selection regions (BSR) for the insertion enthesis of *opponens digiti minimi* on the right fifth metacarpal, before (A) and after (B) applying the "Discrete Curvatures" filter. Scale is not respected.

STEP CASE

7. Costoclavicular ligament (CCL) 1 step

For the attachment site of the costoclavicular ligament (clavicle):

7.1 **Case 7 Steps 7-12 (summary):** The 3D surface model of the clavicle should be viewed from an inferior-medial perspective, as shown in Fig. 15, with the medial end oriented upwards and the bone positioned upright. In this view, the broader selection region (BSR) should encompass the entire typically oval-shaped attachment surface area, which is usually outlined by lines and edges displayed in blue shades after applying the “Discrete Curvatures” filter (see Fig. 15). Ensure that you include all clearly connected blue lines and edges around the oval-shaped area. For example, in Fig. 15, the oblique blue-colored lines extending to the right should be included, as they are linked to the oval-shaped structure. However, the observer must exclude any projecting blue structures not connected to the outline (e.g., those indicated by the two black arrows in the figure). In cases where there are no lines or edges of considerable size within the attachment area (i.e., no projections of at least 5 mm in maximum length), or if the only present alterations appear to be taphonomic and/or pathological in nature (see instructions in Case 1 Step 11), it is recommended to record an area measurement of “0” (as in the previous example of the *opponens digiti minimi*), unless enthesopathies are of interest for the intended analysis/study.

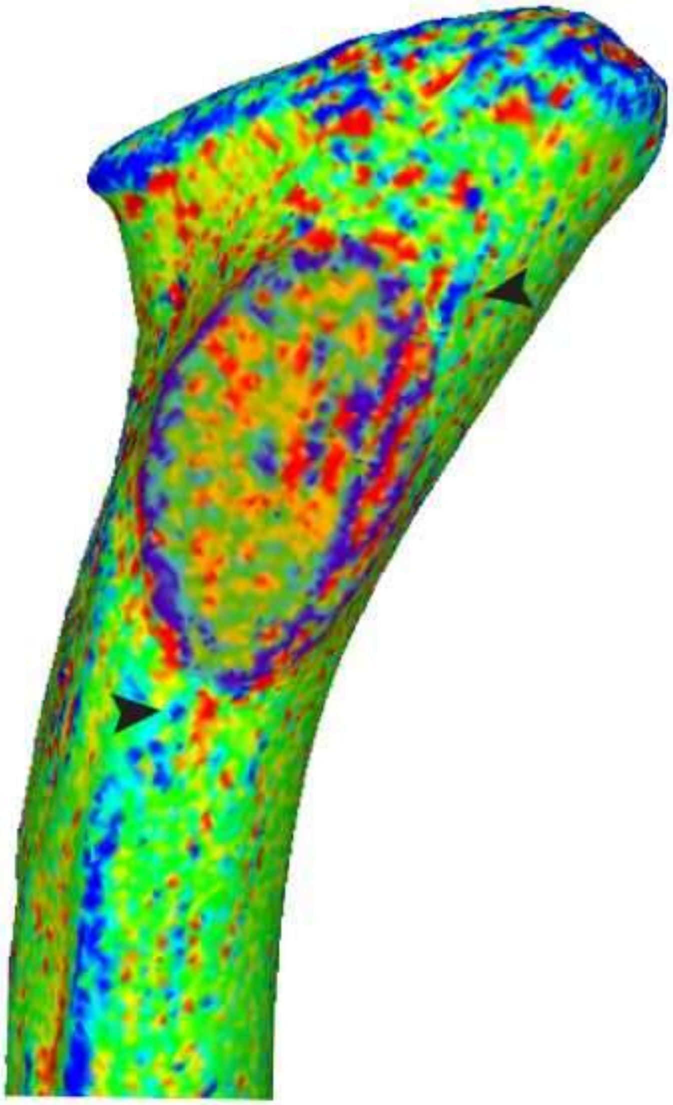

Fig. 15: Selection perspective (SP) and broader selection region (BSR) for the attachment site of the costoclavicular ligament on the clavicle. The view is from an inferior-medial perspective, with the medial end of the clavicle facing upward. The "Discrete Curvatures" filter has been applied in the software Meshlab. Scale is not respected.

STEP CASE

8. Deltoid (DT) 1 step

For the insertion site of the deltoid muscle (i.e., the deltoid tuberosity of the humerus):

7.1 **Case 8 Steps 7-12 (summary):** In the 3D model of the humerus, the deltoid tuberosity should be viewed as shown in Fig. 16, from a lateral view, looking towards the proximal end of the bone, with the medial side

of the humeral head facing upward and away. An oblique angle of approximately 45 degrees is recommended to better distinguish projecting lines and edges within the tuberosity, which will appear in blue shades after applying the “Discrete Curvatures” filter. The broader selection region (BSR) should encompass the entire elevated area of the deltoid tuberosity visible from this perspective, which often carries a combination of sharp blue lines and red depressions (Fig. 16). Any projections or depressions presenting taphonomic and/or pathological characteristics should be excluded using the marker tool and the right mouse button in Meshlab, following the previous recommendations (refer to Case 1, Step 11).

In case that the elevation of the deltoid tuberosity also extends beyond the usual lateral aspect of the bone, you must additionally rotate the bone anteriorly and posteriorly along the same axis and perspective to include these elevated areas in the selection. If that happens, then ensure that these rotation steps are always applied consistently across all specimens in the sample (in the same order and direction) and that this procedure is documented in detail in the study.

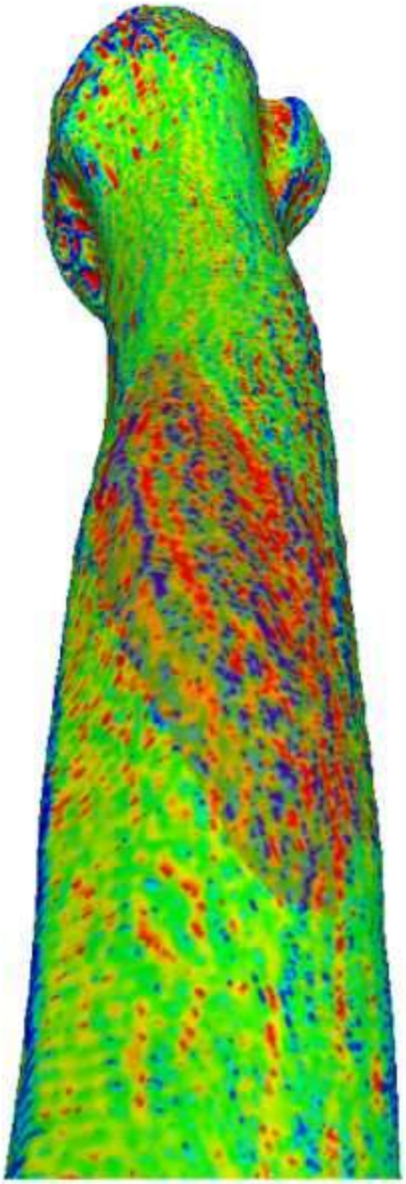

Fig. 16: Selection perspective (SP) including the highlighted broader selection region (BSR) for the insertion site of the deltoid muscle on the humerus (i.e., the deltoid tuberosity). The view is oblique, at approximately 45 degrees, focusing on the latero-proximal part of the bone. The medial aspect of the humeral head is oriented upward and away. The "Discrete Curvatures" filter has been applied in Meshlab. Scale is not respected.

STEP CASE

9. Calcaneal tuberosity (CTUB) 1 step

For the insertion site of the Achilles tendon (calcaneus):

7.1 **Case 9 Steps 7-12 (summary):** For this enthesis, the 3D model of the calcaneus should be viewed from a posterior upright perspective, with the superior aspect of the bone facing upward. The exact angle should be adjusted to minimize the visibility of all anterior structures of the bone, ideally revealing only the near-rectangular posterior aspect of the bone, as shown in Fig. 17. From this perspective, the broader selection region (BSR) must encompass the entire superior two-thirds of the visible area (Fig. 17A), excluding the inferior one-third. Additionally, the most superior border of this region (indicated by the four upper black arrows in the example of Fig. 17A) should be deselected. Subsequently, the bone should be rotated first laterally (Fig. 17B up) and then medially (Fig. 17B down). From these two angles, any areas showing distinguishable taphonomic or pathological alterations (examples marked in Fig. 17B by black arrows) should also be deselected, in line with the detailed recommendations of Case 1 Step 11.

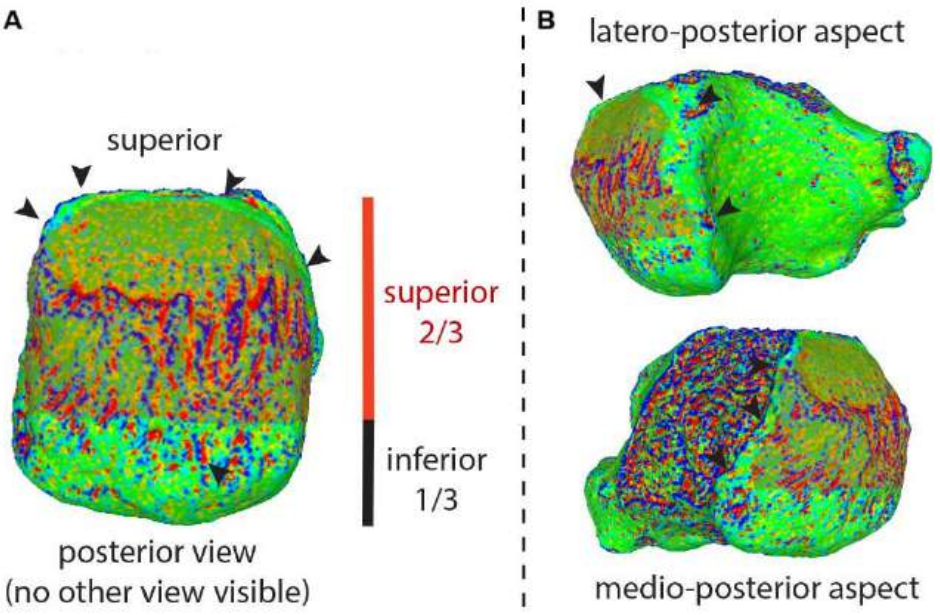

Fig. 17: Selection perspective (SP) showing the highlighted broader selection region (BSR) for the calcaneal insertion site of the Achilles tendon (A). The figure also includes additional views (B) used to confirm that no taphonomic or pathological alterations were accidentally selected beyond the BSR. The “Discrete Curvatures” filter has been applied in Meshlab for both panels. Scale is not respected.

protocols.io
